# Comparison of Synthetic Pathways for Obtaining Fluorescent Nanomaterials Based on Halloysite and Carbon Dots for Potential Biological Sensing

**DOI:** 10.3390/ijms25105370

**Published:** 2024-05-14

**Authors:** Marina Massaro, Giuseppe Cinà, Giuseppe Cavallaro, Giuseppe Lazzara, Alessandro Silvestri, Raquel de Melo Barbosa, Rita Sànchez-Espejo, César Viseras-Iborra, Monica Notarbartolo, Serena Riela

**Affiliations:** 1 Dipartimento di Scienze e Tecnologie Biologiche, Chimiche e Farmaceutiche (STEBICEF), University of Palermo, Viale delle Scienze, Parco d’Orleans II, Ed. 16-17, 90128 Palermo, Italy; marina.massaro@unipa.it (M.M.); giuseppe.cina05@unipa.it (G.C.); monica.notarbartolo@unipa.it (M.N.); 2 Dipartimento di Fisica e Chimica E. Segrè (DiFC), University of Palermo, Viale delle Scienze, Parco d’Orleans II, Ed. 17, 90128 Palermo, Italy; giuseppe.cavallaro@unipa.it (G.C.); giuseppe.lazzara@unipa.it (G.L.); 3 Consorzio Interuniversitario Nazionale per la Scienza e Tecnologia dei Materiali (INSTM), 50121 Firenze, Italy; 4 Center for Cooperative Research in Biomaterials (CIC biomaGUNE), Basque Research and Technology Alliance (BRTA), Paseo de Miramon 194, 20014 Donostia-San Sebastián, Spain; alessandro.silvestri@unive.it; 5 Department of Pharmacy and Pharmaceutical Technology, School of Pharmacy, University of Seville, C/Professor García González 2, 41012 Sevilla, Spain; rdemelo@us.es; 6 Department of Pharmacy and Pharmaceutical Technology, Faculty of Pharmacy, University of Granada, Campus Universitario de Cartuja, 18071 Granada, Spain; ritamsanchez@ugr.es (R.S.-E.); cviseras@ugr.es (C.V.-I.); 7 Andalusian Institute of Earth Sciences, CSIC-UGR, 18100 Armilla, Granada, Spain; 8 Dipartimento di Scienze Chimiche (DSC), University of Catania, Viale Andrea Doria 6, 95125 Catania, Italy

**Keywords:** halloysite, carbon dots, fluorescent sensors, Fe^3+^ ions detection, cellular uptake

## Abstract

Recently, fluorescent sensors have gained considerable attention due to their high sensitivity, low cost and noninvasiveness. Among the different materials that can be used for this purpose, carbon dots (CDs) represent valuable candidates for applications in sensing. These, indeed, are easily synthesized, show high quantum yield and are highly biocompatible. However, it was pointed out that the photoluminescence properties of these nanomaterials are strictly dependent on the synthetic and purification methods adopted. The presence of halloysite nanotubes (HNTs), a natural, low cost and biocompatible clay mineral, has been found to be efficient in obtaining small and highly monodispersed CDs without long and tedious purification techniques. Herein, we report the comparison of synthetic pathways for obtaining halloysite-N-doped CDs (HNTs-NCDs) that could be used in biological sensing. One was based on the synthesis of N-doped CDs by a bottom-up approach on HNTs’ surface by a MW pyrolysis process; the other one was based on the post-modification of pristine N-doped CDs with halloysite derivatives. The evaluation of the best synthetic route was performed by different physico-chemical techniques. It was found that the bottom-up approach led to the formation of N-doped CDs with different functional groups onto the HNTs’ surface. This evidence was also translated in the different fluorescence quantum yields and the existence of several functional groups in the obtained materials was investigated by potentiometric titrations. Furthermore, the ability of the synthesized nanomaterials as sensors for Fe^3+^ ions detection was assessed by spectroscopic measurements, and the cellular uptake was verified by confocal/fluorescence microscopies as well.

## 1. Introduction

Recently, fluorescence-based sensing has gained considerable attention because of its numerous advantages associated with it, such as short response time, excellent sensitivity and, most important, low cost [1,2]. Different fluorescent sensory materials have been synthesized, including metal–organic frameworks, organic dyes, quantum dots and so on [3,4,5]. Although the advantages of these materials are remarkable, their use is not without problems. In this context, carbon dots (CDs), because of their economical synthesis approaches, high fluorescent quantum yield, excellent brightness and biocompatibility, have been efficiently employed in sensor applications [6,7,8,9,10,11,12]. CDs can be easily prepared by exploiting environmentally friendly, cost-effective and energy-efficient one-step synthetic procedures, such as the microwave irradiation method. In this approach, a conversion of larger carbon compounds into smaller components is carried out by pyrolysis processes [13]. Depending on the experimental conditions adopted, the choice of precursors, pre-treatment or post-treatment, it is possible to obtain CDs with different sizes and tunable photoluminescence properties for several applications [4]. In addition, recently, it was raised that the luminescence properties of CDs are strictly affected by artifacts that could be due to the presence of molecular side impurities [14,15,16]. Thus, it is necessary to improve the purification methods to achieve the highest performance in the final materials. Up to now, the common strategies adopted for the CD purifications are based on low cut-off dialysis, which, unfortunately, is not always efficient. So other alternative purification techniques have been adopted [13]. 

In recent years, our groups synthesized CDs by a bottom-up approach, in which the carbon source was previously linked to an inorganic support source such as halloysite nanotubes (HNTs). The presence of HNTs and the low loading of the organic molecules on their surface allowed us to obtain small and highly monodispersed CDs with interesting photoluminescence properties [17].

HNTs are clay minerals with a chemical formula of Al_2_Si_2_O_5_(OH)_4_ × *n*H_2_O, which keeps attracting notable attention for their use in several fields [18,19,20,21,22]. In particular, HNTs have been more often used for biomedical applications [23,24] since they show the intrinsic capability to cross cellular membranes, localizing themselves in the perinuclear region [25,26]. The different chemical composition of the tube surface (Si-O-Si groups on the external surface and Al-OH groups on the inner one) and the presence of an empty lumen are important features of achieving selective HNTs’ chemical modification [27] for the delivery of active species, such as antioxidants [28], PNA [20,29], chemotherapeutic drugs [30,31] and so on [32]. Recently, the loading of a halochromic probe into HNTs was a useful strategy for the development of fluorescent nanomaterials for the detection of cancer cells [33].

In the present work, we report the synthesis and characterization of fluorescent materials based on N-doped CDs and HNTs (HNTs-NCDs) as potential systems for biological sensing. To find the best experimental conditions to obtain luminescent HNTs-NCDs nanomaterials, two different synthetic routes were investigated: (i) a series of HNTs-NCDs were obtained by pyrolysis, starting from HNTs-itaconic acid in the presence of passivant agents [17]. Itaconic acid was chosen as a natural renewable carbonaceous source since it arises from the fermentation of carbohydrates, such as glucose, using *Aspergillus terreus* [34]; (ii) HNTs-NCDs nanomaterial was obtained by post-modification of NCDs [13] by reaction with HNTs-COOH or HNTs-NH_2_ derivatives. In each synthetic strategy adopted, the presence of HNTs could enhance the cellular uptake of the final nanomaterials obtained, and the presence of their empty lumen could be useful for the simultaneous delivery of active species. 

All nanomaterials obtained were characterized by several physico-chemical techniques and the photoluminescence properties were investigated and compared as well. 

Once the most optimal synthetic route for the most promising nanomaterial was assessed, the cellular uptake and sensing capacity by means of confocal/fluorescence microscopies and fluorescence titration with Fe^3+^ ions were also evaluated. Fe^3+^ ions were chosen since they are involved in many biological processes; for example, their deficiency leads to anemia, while their excessive intake causes different kinds of diseases, such as cancer [35], Parkinson’s syndrome [36] and Alzheimer’s disease [37]. The obtained results showed that the bottom-up approach seems to lead to the formation of CDs with different functional groups onto HNTs’ surface. Furthermore, because of the presence of HNTs’ empty lumen, the developed nanomaterials could, in the future, allow for the simultaneous delivery of active species with synergistic actions.

## 2. Results and Discussion

### 2.1. Synthesis of N-Doped CDs Covalently Linked to HNTs External Surface

**Scheme 1:**
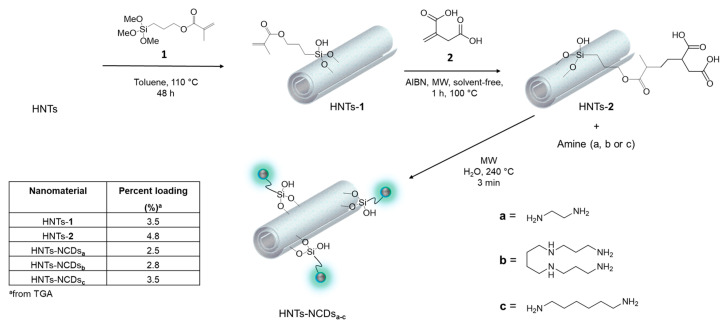
Schematic representation of the synthesis of N-doped CDs directly linked onto HNTs-NCDs_a–c_ nanomaterials. The inset shows the organic portion loaded onto the HNTs’ surface after each synthetic step, estimated by TGA.

This approach is based on a three-step procedure as depicted in Scheme 1. 

Firstly, the external surface of HNTs was modified by grafting 3-methacryloxypropyltrimethoxy silane following the synthetic strategy reported elsewhere [38]. As a result, HNTs-**1** nanomaterial was obtained with a degree of functionalization (determined by thermogravimetric analysis, TGA) of 0.30 mmol g^−1^, which corresponds to a loading percent of 3.5 wt%, in agreement with modification of the few Si-OH groups present at HNTs external surface. Then, HNTs-**1** was used for the linkage of itaconic acid (**2**) by AIBN-catalyzed ene–ene reaction in solvent-free conditions, under microwave irradiation, affording the HNTs-**2** which showed a degree of functionalization of 0.18 mmol g^−1^. It should be noted that, based on the stoichiometric ratio, the molar ratio between the -ene groups and the carboxylic acid is 2:1, indicating that full modification did not occur, probably because of steric hindrance. This could be promising from the perspective of improving nanomaterial cellular uptake. Thus, the nanomaterial was subjected to pyrolysis mediated by MW irradiation with a heating time of 3 min, at 240 °C, using water as the reaction medium, in the presence of amine as a passivating agent. Three different amines were used to achieve a rational design of fluorescent nanomaterials with tunable photoluminescence properties, nitrogen and carboxylic group content, namely ethylenediamine (a), spermine (b) and hexamethylenediamine (c). After work up, the solid nanomaterials, HNTs-NCDs**_a_**_–**c**_, were collected by centrifugation, and the obtained powders were extensively washed with ethanol. 

#### Characterization of HNTs-NCDs_a–c_ Nanomaterials

As mentioned above, the amount of organic moiety grafted to the HNT surfaces at each synthetic step was determined by TGA. The TG data normalized by the water content are reported in Figure S1. Besides the typical mass loss of HNTs at ca. 450–550 °C, which corresponds to the expulsion of the interlayer water molecules [39], some additional weight losses in the range of 250–350 °C are present due to the degradation and volatilization of organic matter. By analyzing the residual matter at 800 °C, it was possible to calculate the percent loading of carboxylic acid onto HNTs for the HNTs-**2,** which corresponds to 4.8 wt%. After the pyrolysis process, the loading amount was reduced in each case investigated according to the formation of N-doped CDs onto HNTs’ external surface; the percent loading of N-doped CDs onto HNTs is reported in Scheme 1.

**Figure 1:**
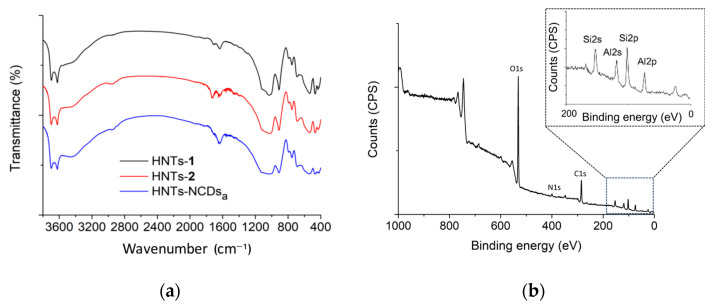

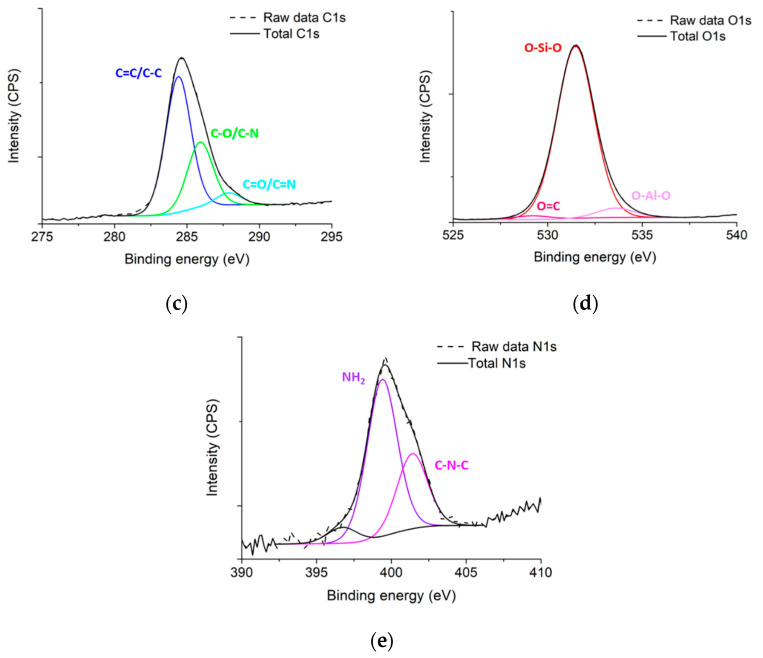
(**a**) FT-IR spectra of HNTs-**1**, HNTs-**2** and HNTs-NCDs**_a_**; (**b**) XPS survey of HNT-NCDs**_a_**; (**c**) deconvoluted C 1s spectrum; (**d**) deconvoluted O 1s spectrum; (**e**) deconvoluted N 1s spectrum.

The successful synthesis was also verified by FT-IR and XPS spectroscopies. In Figure 1a, the FT-IR spectra of HNTs-**1**, HNTs-**2** and HNTs-NCDs**_a_** are reported. As it is possible to observe, after the condensation reaction between the -ene groups onto HNTs and itaconic, the FT-IR spectrum of HNTs-**2** nanomaterial showed typical vibration features of both precursors [40]. As it is possible to observe, FT-IR spectra of HNTs-**1** and HNTs-**2** are clearly observable in the bands at ca. 3602 and 3690 cm^−1^ corresponding to the O–H stretching of the inner hydroxyl groups and outer surface hydroxyl groups, respectively, of HNTs and the broad signal at 1640 cm^−1^ attributable to the H–O–H bending of H-bonded water on the halloysite structure, which corresponds to the broad O–H stretching signal at 3550 cm^−1^. In addition, in both spectra, a further stretching band at ca. 1700 cm^−1^ is present due to the stretching vibration of esters and carboxylic groups of methacryloxy and itaconic acid moieties. After the pyrolysis process, the HNT-NCDs**_a_** spectrum showed some differences, indicating that a change in the structure of the nanomaterial has occurred. In particular, the disappearance of the vibration band at ca. 1790 cm^−1^ related to the stretching of -COOH groups and the presence of new vibration bands in the range 1600–1400 cm^−1^ related to C–N and C=N/C=C functional groups of N-doped CDs can be clearly observed. Similar considerations can be made for the synthesized HNTs-NCDs**_b_**_–**c**_ nanomaterials (Figure S2).

The different functional groups present at the surface of the different HNTs-NCDs**_a–c_** nanomaterials were also proved by XPS measurements. In Figure 1b, the XPS survey spectrum for HNT-NCDs**_a_** is reported. From the survey spectrum of HNT-NCDs**_a_**, C, N and O atoms are detected with peaks at 285.04 eV (C 1s), 399.29 eV (N 1s) and 531.38 eV (O 1s), respectively. In addition, in the HNT-NCDs_a_, representative peaks for the Al 2s, Al 2p, Si 2s and Si 2p are also observed, attributed to the presence of HNTs. Similar results are obtained for synthesized HNTs-NCDs**_b–c_** (Figures S3 and S4).

The XPS high-resolution spectrum of the C 1s core (Figure 1c) was deconvoluted into two components corresponding to sp^2^ (C=C) at 284.4 eV and C-O at 285.8 eV. The O 1s spectrum was deconvoluted into three peaks centered at 528.8, 531.4 and 533.6 eV corresponding to C=O, O-Si-O and O-Al-O groups, respectively (Figure 1d). The N 1s spectrum was deconvoluted into two peaks centered at 399.1 and 400.9 eV related to NH_2_ and C–N–C, respectively (Figure 1e). 

The XPS results show that all nanomaterials synthesized possess several functional groups on their surface, but with different contents (Table S1) depending on the amines used, as previously reported. 

The aqueous mobility of all synthesized nanomaterials was investigated by Dynamic Light Scattering (DLS) measurements, and the obtained results are reported in Table 1. The introduction of carboxylic acids in HNTs-**2** slows the aqueous diffusion of the HNTs as shown by the increased values of apparent hydrodynamic diameters in comparison to that of pristine HNTs. These results might be due to hydrophobic interactions occurring between the organic moieties covalently linked onto the outer surface of the halloysite. Similar observations were found for HNTs with a selective modification of the external surface by exploiting both covalent and supramolecular interactions [17]. The presence of carboxylic groups on the external surface slightly enhances the negative charge of halloysite, as evidenced by the ζ-potential data (Table 1). As concerns HNTs-NCDs, we estimated ζ-potential values between −25.7 ± 1.9 mV (HNTs-NCDs**_a_**) and −18.2 ± 1.7 mV (HNTs-NCDs**_b_**). Moreover, we observed that the HNTs-NCDs nanomaterial presents lower hydrodynamic diameters compared to the precursor, highlighting that the microwave-assisted pyrolysis step increases the aqueous mobility of the modified nanotubes. Specifically, we detected the lowest hydrodynamic diameter for the HNTs-NCDs**_b_** sample (433 ± 64 nm), while the slowest aqueous dynamics was observed for HNTs-NCDs**_a_**, which presents a hydrodynamic diameter equal to 523 ± 35 nm. 

**Figure 2:**
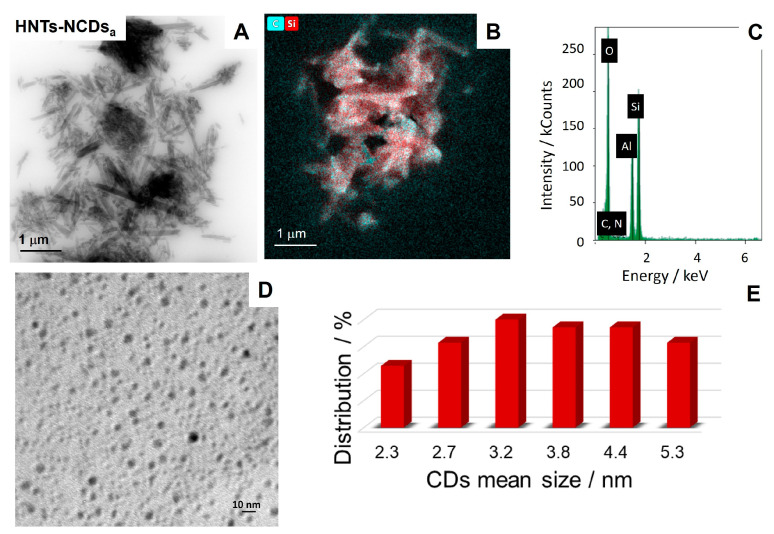
(**A**) TEM of the HNTs-NCDs**_a_** nanomaterial; (**B**) EDX elemental mapping images. (**C**) EDS analysis, (**D**) TEM images of N-doped CDs obtained after hydrolysis of HNTs-NCDs**_a_**; (**E**) statistical analysis (*n* = 70).

The morphology of HNTs-NCDs**_a_**, chosen as the model, was investigated by high-angle annular dark field scanning transmission electron microscopy (HAADF-STEM), which showed that the nanomaterial preserved the tubular structure of halloysite after the pyrolysis process (Figure 2). It is also possible to observe that the tubes appear agglomerated as a consequence of the existence of some attractive interactions among the different N-doped CD units present at the external surface, in agreement with that reported in the literature [17]. Energy Dispersive X-ray measurements (EDX) show the presence of C and N atoms related to the organic portions in addition to the typical elements of the clay, namely, Si, Al and O atoms (Figure 2C). From elemental mapping extrapolated from EDX, it is possible to conclude that the N-doped CD units are uniformly distributed onto HNTs, as highlighted by the spots related to the C and N atoms (Figure 2B).

Furthermore, in order to prove the formation of small and highly monodispersed N-doped CDs, the CDs attached to HNTs’ external surface were hydrolyzed in slightly acid conditions and collected by centrifugation. The supernatant solution was analyzed by TEM, which shows the presence of carbon dots with a quasi-spherical morphology and a rather homogeneous size distribution (Figure 2D,E). By statistical analysis, the average size of these N-doped CDs was estimated to be as large as 3.7 ± 0.9 nm.

### 2.2. Post-Modification of N-Doped CDs by Modified HNTs 

**Scheme 2:**
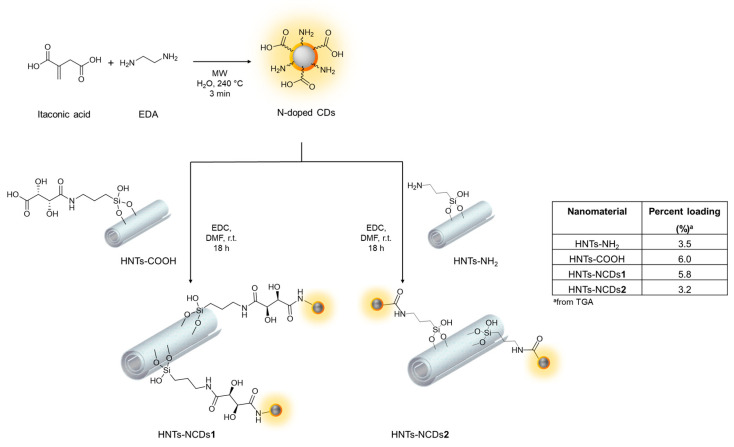
Schematic representation of the synthesis of HNTs-NCDs**1** and HNTs-NCDs**2** nanomaterials. The inset shows the organic portion loaded onto the HNTs’ surface after each synthetic step, estimated by TGA.

N-doped CDs were prepared according to the synthetic procedure reported in Scheme 2 by reacting itaconic acid (**2**) with ethylenediamine under MW irradiation. These precursors were chosen since it is reported that they produce N-doped CDs with enhanced photoluminescence properties [34]. After purification by gel filtration chromatography [13], CDs with dimensions of ca. 1.2 ± 0.2 nm were obtained. Successively, N-doped CDs were post-modified by reaction with HNTs derivates with amine or carboxylic acids terminal groups, namely HNTs-NH_2_ and HNTs-COOH. The condensation reaction was carried out in the presence of EDC to afford the HNTs-NCDs**1** and HNTs-NCDs**2** nanomaterials, according to the HNTs precursor used. 

HNTs-NCDs**1** and HNTs-NCDs**2** nanomaterials were characterized by FT-IR spectroscopy, TGA, XPS, DLS and ζ-potential measurements, and the morphology was investigated by HAADF/STEM images.

Similar to that observed in the case of HNTs-NCDs**_a_** nanomaterial, the FT-IR spectra of the HNTs-NCDs**1** and HNTs-NCDs**2** showed the typical vibration bands of HNTs and those related to the organic moieties grafted on their external surface. They are, indeed, clearly observable in the bands in the range 1600–1400 cm^−1^ related to C–N, C=N/C=C functional groups of N-doped CDs (Figure S5).

TGA shows that both HNTs-NCDs**1** and HNTs-NCDs**2** present three major mass losses in the range 25–150 °C, 200–400 °C and 450–550 °C that can be attributed to the evaporation of the water molecules physically adsorbed onto the nanomaterials, thermal decomposition of organic compounds and expulsion of the interlayer water molecules of halloysite, respectively (Figure S6). It should be noted that the mass loss at 200–400 °C is larger for HNTs-NCDs**1** (5.8 wt%) with respect to that of HNTs-NCDs**2** (3.2 wt%). On this basis, we can affirm that the CD load is higher for HNTs-NCDs**1**, according to the loading percent of the HNTs-COOH and HNTs-NH_2_ precursors.

Comparing the XPS analyses of HNTs-NCDs**1** and HNTs-NCDs**2** with the one of HNTs-NCDs**_a_** several differences are present, in terms of both functional groups (Figure S7) and content (Tables S1 and S2). This could indicate that the two synthetic strategies influence the chemical features of the final CDs. 

Dynamic Light Scattering measurements evidenced that the CDs linkage onto the halloysite external surface enhances the aqueous mobility of HNTs-NCDs**1** and HNTs-NCDs**2**, as shown by the decrease in the apparent hydrodynamic diameter. Specifically, we determined hydrodynamic diameters of 380 ± 37 nm and 422 ± 46 nm for HNTs-NCDs**1** and HNTs-NCDs**2**, respectively. Both values are lower compared to those reported in Table 1. Conversely, pristine N-doped CDs dispersed in water showed a hydrodynamic diameter of 749 ± 39 nm as a consequence of the diffusion of aggregates of carbon dots in aqueous media. The existence of aggregation phenomena indeed was recently reported by Yu, Chen et al. [41], who, similarly, detected a hydrodynamic diameter of ca. 1000 nm for aggregates of nitrogen–sulfur co-doping carbon dots. 

Regarding the surface charge of the nanomaterials, ζ-potential measurements showed that both HNTs-NCDs**1** and HNTs-NCDs**2** possess ζ-potential values (−16.1 ± 1.7 mV and −14.7 ± 0.4 mV, for HNTs-NCDs**1** and HNTs-NCDs**2**, respectively) close to that of pristine HNTs (−18 mV). Noteworthy, this value is quite different from the one of HNTs-NCDs**_a_** (ca. −26 mV). These results might indicate that some protonable/deprotonable groups on the CD surface disappear during their post-modification; indeed, the ζ-potential value of the pristine CDs is −22 ± 3 mV.

TEM investigations on pristine N-doped CDs (Figure S8) showed that they possess a typical round shape with a mean size of ca. 1.2 ± 0.2 nm [13,34].

**Figure 3:**
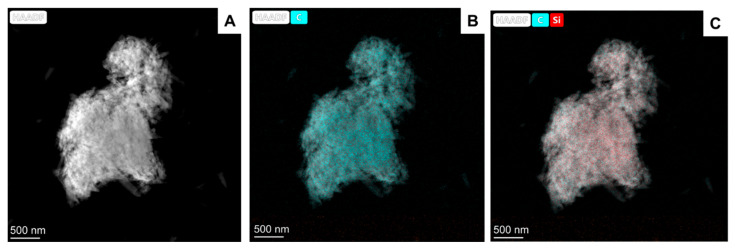
(**A**) HAADF/STEM image of HNTs-NCDs**1**; (**B**,**C**) EDX elemental mapping images.

After their linkage on HNTs derivatives (HNTs-NH_2_ or HNTs-COOH), the morphology of the resulting nanomaterials compared to the one of HNTs-NCDs**_a_**_–**c**_ is quite different (Figure 3 and Figure S9). It was indeed observed the formation of big aggregates of HNTs, where the tubes seem to be linked together. Thus, it was hypothesized that this peculiar morphology could arise from the synthetic procedure; indeed, during the condensation reaction, different CD units could be bridged among different HNTs by amide groups.

### 2.3. Photoluminescence Properties of HNTs-NCDs 

UV–*vis* absorption and photoluminescence (PL) were used to investigate the optical characteristics of all CD-based nanomaterials synthesized. Aqueous dispersions of all nanomaterials obtained (HNTs-NCDs**_a–c_**, HNTs-NCDs**1** and HNTs-NCDs**2**) showed a bright blue luminescence under UV light (325 nm), as already reported for similar systems.

**Figure 4:**
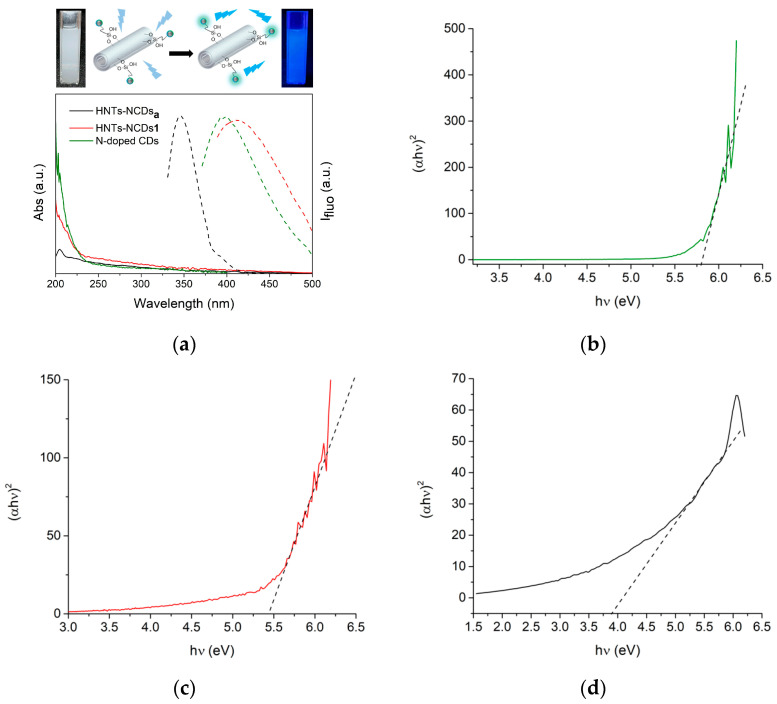
(**a**) Absorption and PL emission spectra of N-doped CDs, HNTs-NCDs**1** and HNTs-NCDs**_a_** (0.5 and 0.02 mg mL^−1^, respectively) in water ([CDs] = 0.04 mg mL^−1^) (the inset shows the PL properties of the HNTs-NCDs**_a_** irradiated at 325 nm), (**b**–**d**) Tauc plot of (**b**) N-doped CDs, (**c**) HNTs-NCDs**1** and (**d**) HNTs-NCDs**_a_**.

The UV-*vis* spectra of HNTs-NCDs and pristine N-doped CDs**_a_** nanomaterials, as shown in Figure 4a and Figure S10, exhibited a wide absorption primarily in the UV region (230–350 nm), with a tail extending into the visible range, with an absorption band at ca. 230 nm. A proper comparison among the spectra of pristine N-doped CDs and the HNTs-NCDs-based nanomaterials obtained by the two synthetic strategies highlighted some differences in the wavelength at which the absorption starts in the visible region [42]. To shed light on the origin of these differences, the energy band gap values (E_g_) were calculated by means of the Tauc plot (Figure 4b–d). As it is possible to infer, the pristine N-doped CDs showed a value of E_g_ = 5.8 eV, the HNTs-NCDs**1** and HNTs-NCDs**2** nanomaterials (Figure S10c) have E_g_ value of ca. 5.4 eV, whereas HNTs-NCDs**_a_** has E_g_ value of ca. 3.90 eV. As already reported [42], low band gap values can be related to the generation of compensatory energy states caused by the presence of different functional groups onto HNTs’ surface in HNTs-NCDs**_a_** nanomaterials that are missing both in the pristine N-doped CDs and in HNTs-NCDs**1** and HNTs-NCDs**2**. This could be the reason for the shift in the conduction band edges.

**Figure 5:**
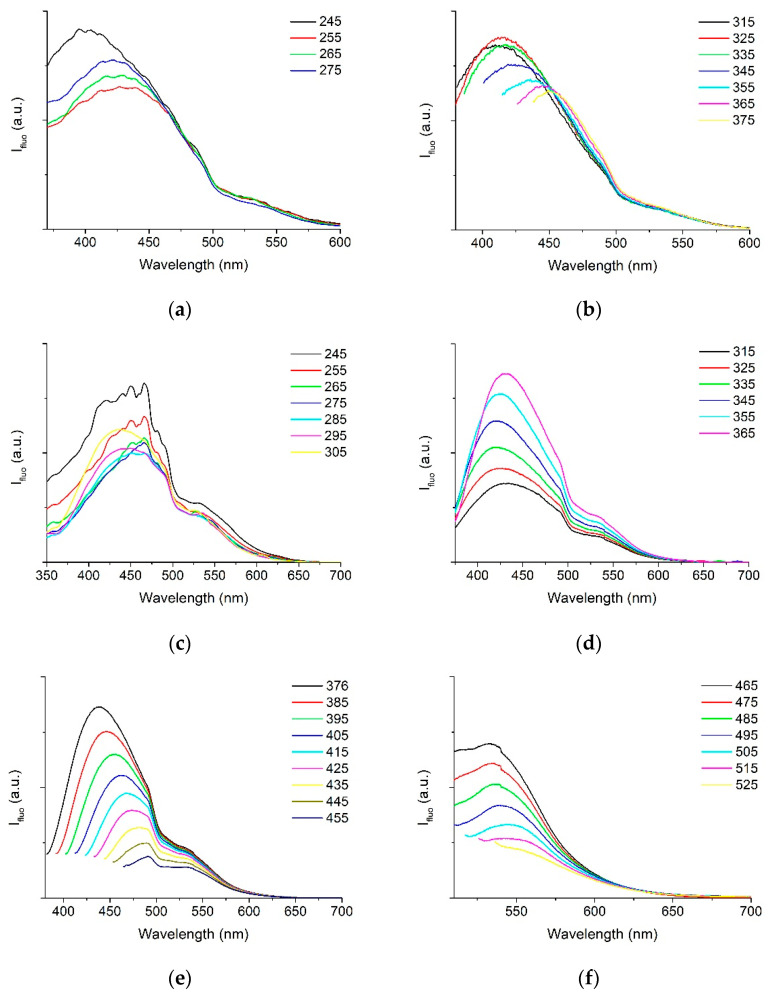
PL spectra in (**a**,**b**) solution and (**c**–**f**) solid state of HNTs-NCDs**_a_** at different excitation wavelengths.

All nanomaterials showed a wide emission band when excited at 325 nm. In particular, HNTs-NCDs**_a_** showed a maximum emission band centered at ca. 420 nm, which is red-shifted of ca. 20–40 nm in the emission spectra of pristine N-doped CDs and HNTs-NCDs**1** (400 nm and 380 nm for N-doped CDs and HNTs-NCDs**1**, respectively, Figure S4). These differences could be explained on the basis of the different optical band gap values calculated above. Furthermore, all HNTs-NCDs nanomaterials synthesized showed the typical excitation-wavelength emission both in solution and in the solid state as a result of the different functional groups present on their surface (Figure 5 and Figures S11–S16). The relative fluorescence quantum yields (FLQY) of the synthesized nanomaterials excited by UV light at 325 nm are reported in Table 2, calculated by selecting quinine sulfate as standard. As it is possible to observe, the HNTs-NCDs**_a_** nanomaterial (entry 2) showed a FLQY value close to that of pristine N-doped CDs (entry 1), indicating that the PL properties of CDs were preserved by adopting this synthetic procedure. Conversely, the linkage of CDs onto HNTs’ surface, in HNTs-NCDs**1** and HNTs-NCDs**2**, led to the synthesis of fluorescent nanomaterials with low FLQY. Since the quantum yield is strongly dependent on the -NH_2_ groups content onto the nanomaterials [43], probably they reacting during the condensation reaction were lost.

To further prove this hypothesis, acid–base potentiometric titrations were performed to verify the presence of protonable/deprotonable groups (-COOH or -NH_2_) onto the CDs surface. 

**Figure 6:**
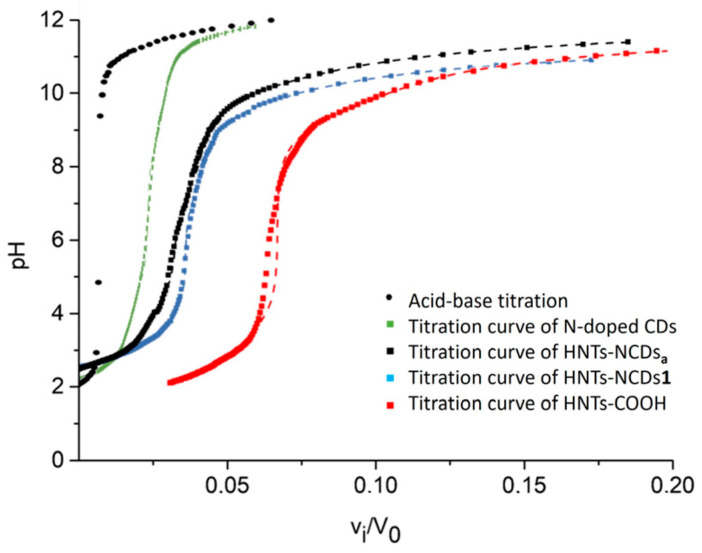
Potentiometric titration of N-doped CDs, HNTs-NCDs**_a_**, HNTs-NCDs**1** and HNTs-COOH. The first black trend represents the titration of HCl (0.1 M) solution with NaOH 0.25 M for comparison.

In particular, aqueous dispersions of HNTs-NCDs**_a_**, pristine N-doped CDs or HNTs-NCDs**1** nanomaterials in the presence of an excess of a strong acid (HCl) were titrated with a standard NaOH solution (0.25 M). The obtained results were compared with those of HNTs-COOH titrated under the same experimental conditions. According to the literature, by fitting the experimental titration curves (Figure 6) [44,45], it is possible to determine the number of groups capable of providing protons [46]. The obtained parameters are reported in Table 3.

First of all, it is noteworthy that the aqueous dispersion of each nanomaterial showed different pH values. In particular, the aqueous dispersion of HNTs-NCDs**_a_** nanomaterial and that of pristine N-doped CDs showed pH values of 8.2 and 8.6, for HNTs-NCDs**_a_** and N-doped CDs, respectively, whereas HNTs-NCDs**1** presented a pH value of 4.8, close to those of HNTs-COOH (pH = 4.2). These differences could be explained by the existence of different functional groups at the CD surface, in agreement with ζ-potential measurements, FLQY and antioxidant properties.

Potentiometric data parameters highlighted that the HNTs-NCDs**_a_** nanomaterial shows three different acid groups attributable to the presence of carboxylic and ammonium groups on its surface with different pK values. In particular, the existence of two ${pK}_{{BH}^{+}}$ values could represent the protonation of two different amino groups at the surface of nanomaterial, whereas the pK_a_ one is attributable to the -COOH groups. Pristine N-CDs present two different groups, as well as HNTs-NCDs**1** and HNTs-COOH nanomaterials. From a critical view, it is possible to note that HNTs-NCDs**1** and HNTs-COOH (entries 3–4) present very close values both of pK and content of carboxylic and amino groups. Therefore, one could conclude that the two nanomaterials showed similar groups. Since the only protonable groups on the HNTs-COOH surface are -COOH and the -NH_2_ that did not take part in the condensation reaction (see *infra*), the same groups should be present on HNTs-NCDs**1**. Thus, the post-modification of N-CDs with HNTs by EDC-mediated condensation led to the disappearance of carboxylic and amine groups onto N-CDs, further confirming the hypotheses.

In light of these results, it is possible to conclude that the best synthetic approach for the synthesis of multifunctional fluorescent nanomaterials based on halloysite and carbon dots, in terms of work-up and physico-chemical properties, is a bottom approach. This synthetic approach indeed allows us to obtain nanomaterial where N-CDs are uniform and highly monodispersed without the use of long and tedious purification methods, retaining all luminescence properties of pristine N-CDs. In addition, the presence of HNTs could enhance the cellular uptake of the final nanomaterials, and the presence of an empty lumen could be useful for the simultaneous delivery of active species. 

### 2.4. pH and Solvent Effects on Photoluminescence Properties of HNTs-NCDs Nanomaterials

**Figure 7:**
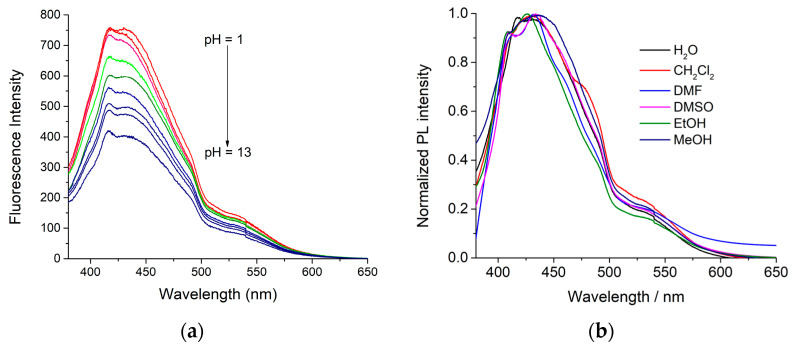
(**a**) PL spectra of HNTs-NCDs**_a_** nanomaterial (0.05 mg mL^−1^) at different pH values; (**b**) Normalized fluorescence spectra of HNTs-NCDs**_a_** nanomaterial in different solvents (λ_ex_ = 365 nm).

To verify the existence of any effects of the pH of the medium on photoluminescence properties of HNTs-NCDs nanomaterials, fluorescence spectra of HNTs-NCDs**_a_**, chosen as models, at different pH were acquired. Figure 7a shows the fluorescence intensity trend as a function of pH, as it is possible to note an increase in the PL intensity with pH decreasing is observed. As reported in the literature, this behavior could be due to the different degrees of protonation of the N-doped CDs at various pH values. Since the degree of protonation increases in acidic conditions, in a pH range between 1 and 4, it is possible to hypothesize that the HNTs-NCDs nanomaterial shows a higher net surface charge, which confers its hydrophilic properties, stability and dispersibility in water, translating into strong fluorescence intensity. Conversely, the maximum emission wavelength was almost the same regardless of solvents, indicating that these have slight effects on the photoluminescence properties.

### 2.5. Fluorescence Sensing of Fe^3+^ Ions

**Figure 8:**
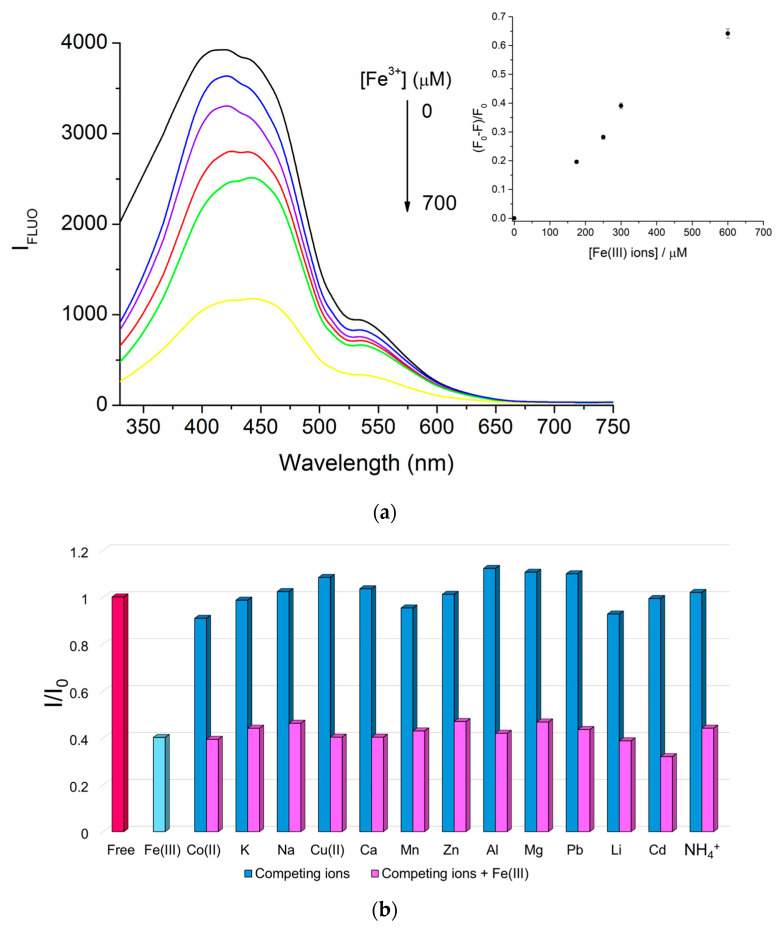
(**a**) Fluorescence intensity of HNTs-NCDs**_a_** (1 mg mL^−1^) (λ_ex_ = 365 nm) upon addition of different concentrations of Fe^3+^ ions. The inset shows the linear plot of HNTs-NCDs**_a_** fluorescence quenching, (F_0_–F)/F, versus Fe (III) concentration at 0–700 μM. Reported are the mean value of two independent assays; (**b**) Relative fluorescent intensities (I/I_0_) of HNTs-CDs (0.05 mg mL^−1^) (λ_ex_ = 365 nm) in the presence of different cations (400 μM) (blue bars) and following treatment of the dispersions with Fe^3+^ ions (400 μM).

To test the feasibility of the developed materials as fluorescent sensors for Fe^3+^ ions; fluorescence spectra of HNTs-NCDs**_a_** aqueous dispersion (1 mg mL^−1^, corresponding to CDs concentration of 0.025 mg mL^−1^) as a function of Fe^3+^ ions concentration were recorded, and the obtained results are reported in Figure 8a. As it is possible to observe, the fluorescence of HNTs-NCDs**_a_** decreases by increasing the Fe^3+^ ions concentration. Furthermore, it was also found that a linear correlation between the quenching efficiency and Fe^3+^ ions concentration exists in a wide pH range. From these preliminary results, it is possible to assume that the developed materials could be promising as sensors in biological fluids. From the linear fitting of the experimental data, it was possible to calculate the LOD that is ca. 24 μM in line with that reported in the literature [47].

To assess the selectivity of the HNTs-NCDs nanomaterial as a fluorescent probe for the detection of Fe^3+^ ions, the change in the HNT-NCDs emission (λ_ex_ = 365 nm) (0.5 mg mL^−1^, corresponding to N-doped CDs concentration of 12.5 μg mL^−1^) in the presence of different ions was evaluated. The obtained results are reported in Figure 8b. As it is possible to observe, a significant quenching of the HNTs-NCDs emission was found only in the presence of Fe^3+^ ions, confirming that the nanomaterial shows selectivity towards this kind of metal ions, that was retained even in the presence of other ions (Figure 8b). 

### 2.6. Biological Evaluation of HNTs-NCDs Nanomaterials

**Figure 9:**
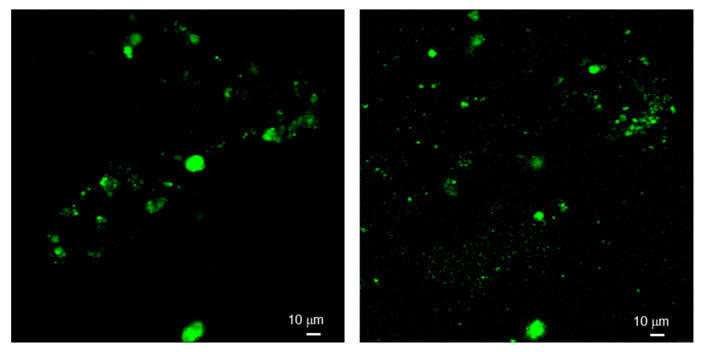
CLSM images of MCF-7 cell lines incubated with HNTs-NCDs**_a_** nanomaterial.

**Figure 10:**
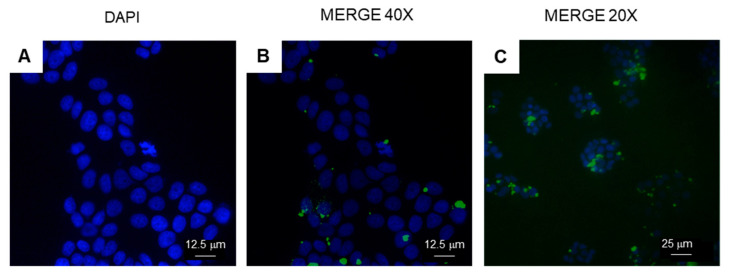
Fluorescence microscopy images of MCF-7 cells treated with HNT-NCDs**_a_** (1 mg mL^−1^) and nuclei fixed stained with DAPI (**A**) 40×, (**B**) merged images 40× and (**C**) merged images 20× of magnification, respectively.

Finally, to explore the cell-imaging potential of the HNTs-NCDs**_a–c_** nanomaterials, their cellular internalization was studied by confocal laser scanning and fluorescence measurements. Preliminary studies showed that the nanomaterials are not cytotoxic in a wide concentration range. The intracellular distribution of HNTs-NCDs**_a_** nanomaterial was investigated by treating MCF-7 cancer cell lines with the nanomaterial (1 mg mL^−1^) for an incubation time of 24 h. As is it possible to observe from both Figure 9 and Figure 10, the HNTs-NCDs**_a_** across cellular membrane mainly localize in the perinuclear region as highlighted by the green fluorescence of the HNTs-NCDs inside cells. Furthermore, close observation of fluorescence images (Figure 9) revealed that a weak fluorescence is also observed in the cell nuclei, as already reported. Therefore, the nanomaterials can also reach the nuclear region and could be promising for future non-viral gene therapy.

## 3. Materials and Methods

All reagents used for nanomaterial synthesis were purchased from Merck (Milan, Italy) and used without further purification. HNTs-NH_2_, HNTs-**1** and CDs_a_ were synthesized as reported elsewhere [17,34,38].

N-CDs_a_ were purified by Gel Filtration chromatography as reported elsewhere [13].

MW-assisted syntheses were performed using a CEM DISCOVER monomode system (CEM Corporation, Matthews, NC, USA) in a closed vessel.

FT-IR spectra (KBr) were acquired by means of an Agilent Technologies Cary 630 FT-IR spectrometer (Agilent Technologies, Santa Clara, CA, USA).

Thermogravimetry analyses were performed by a Q5000 IR apparatus (TA Instruments, New Castle, DE, USA). The measurements were carried out under N_2_ atmosphere (the gas flows were set at 25 and 10 cm^3^ min^−1^ for the sample and the balance, respectively) from 25 to 800 °C. The heating rate was set at 20 °C min^−1^.

XPS measurements were made with a SPECS SAGE HR 100 spectrometer (SPECS, Berlin, Germany) in high vacuum (10^−7^ Pa), equipped with a non-monochromatic X-ray source (Mg) with a Ka line of 1253.6 eV. An electron dilution gun was used to neutralize the charge. The spectra were processed and fitted with Casa XPS (Version 2.3.16 PR!.6.)

DLS and z-potential measurements were acquired by means of a Zetasizer Nano-ZS (Malvern Instruments, London, UK) at 25.0 ± 0.1 °C.

The TEM instrument was an FEI Titan G2 60–300 ultra-high resolution transmission electron microscope (FEI, Lausanne, Switzerland) coupled with analytical electron microscopy (AEM) performed with an energy dispersive X-ray spectroscopy (XEDS) detector. AEM spectra were saved in scanning transmission electron microscopy (STEM) mode with a high-angle annular dark field (HAADF) detector. Elemental maps were also collected using X-rays.

UV-vis measurements were acquired with a Beckman DU 650 spectrometer (Beckman Coulter, Inc., Brea, CA, USA).

Fluorescence measurements, both in solution and in solid state, were performed with a JASCO FP8300 spectrofluorometer (JASCO, Cremella (LC), Italy). For measurements in solution, the excitation and emission slits were set at 5 nm, and spectra were acquired in wavelength intervals ranging between 300 and 700 nm. Solid state spectra were acquired by setting the excitation and emission slits at 5 and 2.5 nm, respectively, in the wavelength interval ranging between 300 and 700 nm.

The quantum yield measurements were performed as reported in the literature [17].

Potentiometric titrations were performed by adding different aliquots (2.5 μL steps) of NaOH solution (0.25 M) to an aqueous dispersion (5 mL) of nanomaterial (20 mg) treated with 0.1 N HCl (300 μL; n_HCl_ = 0.03 mmol). The resulting dispersion was degassed with argon for 10 min before starting the titration. The aqueous NaOH solution was added using a Chemotron microliter syringe, and the pH value was measured using the Crison micro pH 2001 system (Crison, Barcellona, Spain). 

Confocal microscopy images were performed on an Olympus FluoView10i laser scanning confocal microscope (Olympus, Tokyo, Japan) provided with humidity control at 37 °C and using a 10 × 0.3 NA objective lens. Measurements were acquired using laser excitation at 325 nm. The emitted fluorescence was collected in photon counting mode. Spectral detection was performed using a bandwidth of 5 nm and a 3 nm step in the range 420–740 nm. The scan area was 256 × 256 pixels, and the scan rate was 12 μs per pixel.

Fluorescence signals were detected using a fluorescence microscope (Leica, Buccinasco (MI), Italy).

### 3.1. Synthesis

#### 3.1.1. Synthesis of HNTs-COOH

L-tartaric acid (460 mg, 10 equiv.) and 1-ethyl-3-(3-dimethylaminopropyl) carbodiimide (EDC, 62 mg, 10 equiv.) were suspended in anhydrous DMF (15 mL). The suspension was left to stir at rt for 10 min. Then, 0.5 g of HNTs-NH_2_ was quickly added. The dispersion was stirred for 48 h. After this time, the solvent was filtered off, and the powder was washed with H_2_O and CH_2_Cl_2_ and finally dried at 60 °C under vacuum.

#### 3.1.2. Synthesis of HNTs-2

In a closed MW vessel, itaconic acid (50 mg) and HNTs-**1** (500 mg) were weighed, and AIBN, in a catalytic amount, was added. The powder was inserted in the MW apparatus at 100 °C, under constant stirring, for 1 h. The solid was filtered off, rinsed with H_2_O and dried at 60 °C overnight. 

#### 3.1.3. Synthesis of HNTs-NCDs_a-c_

HNT-NCDs**_a–c_** nanomaterials were obtained upon MW irradiation of an aqueous dispersion of HNTs-**2** in the presence of ethylenediamine (EDA), spermine or hexamethylene diamine (1:1 mol mol^−1^). Typically, halloysite-based nanomaterials (100 mg), EDA (150 μL) hexamethylenediamine (50 mg) or spermine (50 mg) and water (460 μL) were heated at 240 °C, at 200 W (the set value) for 240 s in the presence of a catalytic amount of TEA. In the process of MW heating, the dispersion changes color from white to yellow as a result of the formation of N-CDs on the HNTs’ external surface. After this, the solid powder was centrifuged, rinsed several times with EtOH to remove the unreacted reagents and dried at 60 °C under vacuum. 

#### 3.1.4. Synthesis of HNTs-NCDs1

HNTs-COOH (100 mg) and 1-ethyl-3-(3-dimethylaminopropyl) carbodiimide (EDC, 100 mg) were suspended in anhydrous DMF (10 mL). The obtained dispersion was left to stir at r.t. under Ar atmosphere for 10 min. Then, a N-CDs solution in DMF (5 mL) was added. The reaction was left under stirring for 48 h at room temperature. At the end of the reaction, the nanomaterial was washed with H_2_O and dried at 60 °C overnight. 

#### 3.1.5. Synthesis of HNTs-NCDs2

N-CDs and EDC (0.5 mg) were dissolved in anhydrous DMF (15 mL). The solution was stirred under Ar at room temperature for 10 min. Then, 0.1 g of HNTs-NH_2_ was added. The suspension was left to stir at r.t for 48 h. Afterwards, the powder was washed with H_2_O and dried at 60 °C overnight.

#### 3.1.6. Fluorescence Titration for Fe(III) Ions Detection

To a dispersion of HNTs-NCDs_a_ (2 mg/mL), increasing volumes (100–600 µM) of an aqueous Fe(III) ions solutions (1 × 10^−3^ M) were added. The obtained dispersions were degassed for 10 min under Ar flow. Excitation and emission slits were set at 5 nm, and spectra were acquired in wavelength intervals ranging between 330 and 750 nm.

### 3.2. Cell Cultures

The human breast cancer cell line MCF-7 was obtained from ATCC (HTB-22™). The cells were cultured in Dulbecco’s modified Eagle’s medium (DMEM, HyClone Thermo Scientific, Foster City, CA, USA) supplemented with 10% heat-inactivated fetal calf serum, 1 mM sodium pyruvate, 2 mM L-glutamine, 100 μg/mL streptomycin and 100 units/mL penicillin (HyClone Europe) in 5% CO_2_ atmosphere at 37 °C. 

## 4. Conclusions

Herein, we reported the synthesis of fluorescent materials based on halloysite and carbon dots for biological sensing. To find the best experimental route to synthesize CDs with good properties, two different synthetic pathways were investigated. One was based on the synthesis of CDs by a bottom-up approach on HNTs’ surface by an MW pyrolysis process; the other one was based on the post-modification of pristine CDs with halloysite derivatives. The carbonaceous source chosen, in both cases, was itaconic acid since it naturally arises from the fermentation of carbohydrates, such as glucose, using *Aspergillus terreus*. In the first case, to fully exploit the photoluminescence properties, three different amines, as passivant agents, were used. This choice was dictated by the fact that the presence of HNTs could influence the final N-CDs properties, so, at this stage, we would investigate the effects of different amines on the final nanomaterials.

All nanomaterials synthesized were characterized by several techniques aiming to determine the structure, functional groups and morphologies that are dependent on the synthetic route adopted. In addition, spectroscopic measurements both by UV-vis and fluorescence spectroscopies highlighted some differences in the photoluminescence properties, confirmed by different energy band gap values obtained as well. In particular, the bottom-up approach seems to lead to the formation of CDs with different functional groups onto the HNTs’ surface. This evidence was also translated in the different fluorescence quantum yields and the existence of several functional groups in the obtained materials was investigated by potentiometric titrations.

In light of the obtained results, the bottom-up approach is the best synthetic route to obtain multifunctional nanomaterials based on halloysite and carbon dots with enhanced photoluminescence properties. This synthetic approach indeed allows to obtain nanomaterial where CDs are uniform and highly monodispersed without the use of long and tedious purification methods.

Finally, the capability of the nanomaterials to act as fluorescent sensors for Fe^3+^ ions was investigated by fluorescence titration and the cellular uptake was assessed by confocal/fluorescence microscopies finding that the presence of HNTs enhance the cellular uptake of the final nanomaterials. 

From these preliminary results, it is possible to assume that the developed materials could be promising as sensors in biological fluids and as non-viral vectors for gene therapy. As a future perspective, these smart nanomaterials could provide a step ahead in comparison to similar nanomaterials because of the presence of HNTs empty lumen that could allow the simultaneous delivery of active species.

## Figures and Tables

**Table 1:** Average diameters and ζ-potential values of HNTs, HNTs-**2** and HNTs-NCDs_a–c_ nanomaterials.

Nanomaterial	Apparent Hydrodynamic Diameter (nm)	ζ–Potential (mV)
HNTs	295 ± 16	−18.0 ± 1.0
HNTs-**2**	686 ± 57	−25.2 ± 1.0
HNTs-NCDs**_a_**	523 ± 35	−25.7 ± 1.9
HNTs-NCDs**_b_**	433 ± 64	−18.2 ± 1.7
HNTs-NCDs**_c_**	450 ± 55	−23.3 ± 1.0

**Table 2:** Relative fluorescence quantum yield values of N-doped CD-based nanomaterials.

Entry	Nanomaterial	FLQY (%)
1	N-doped CDs	26.0
2	HNTs-NCDs**_a_**	24.0
3	HNTs-NCDs**_b_**	11.0
4	HNTs-NCDs**_c_**	8.0
5	HNTs-NCDs**1**	3.0
6	HNTs-NCDs**2**	3.0

**Table 3:** Calculated parameters obtained by the regression analysis of the experimental titration curves of HNTs-NCDs**_a_**, N-doped CDs, HNTs-NCDs**1** and HNTs-COOH.

Entry	Sample	pH ^a^	pK_a_ ^b^	n_a_/g (mmol g^−1^)	${pK}_{{BH}_{1}^{+}}$ ^c^	nb_1_/g (mmol g^−1^)	${pK}_{{BH}_{2}^{+}}$ ^c^	nb_2_/g (mmol g^−1^)
1	HNTs-NCDs_a_	8.2	4.2	2.6	7.3	2.4	10.3	1.1
2	N-doped CDs	8.6	4.2	4.8	8.2	4.3	/	/
3	HNTs-NCDs**1**	4.8	3.8	3.6	9.9	9.5	/	/
4	HNTs-COOH	4.2	3.5	3.4	9.0	9.6	/	/

^a^ pH of the material aqueous dispersion before titration; ^b^ pK related to the -COOH groups; ^c^ pK related to the amino groups.

## Data Availability

Dataset available on request from the authors.

## Supplementary Materials

The following supporting information can be downloaded at https://www.mdpi.com/article/10.3390/ijms25105370/s1. Reference [48] is cited in Supplementary Materials.
