# Supplementary material for: Comparison of Synthetic Pathways for Obtaining Fluorescent Nanomaterials Based on Halloysite and Carbon Dots for Potential Biological Sensing
Source: Int J Mol Sci. 2024 May 14;25(10):5370. doi: 10.3390/ijms25105370 (PMC11121483; doi:10.3390/ijms25105370)
Supplement: Supplementary file 1 [file ijms-25-05370-s001.zip › ijms-2953431-supplementary.pdf]

Comparison of synthetic pathways for obtaining fluorescent nanomaterials based on halloysite and carbon dots for potential biological sensing

*Marina Massaro,[a] Giuseppe Cinà,[a] Giuseppe Cavallaro,[b,c] Giuseppe Lazzara,[b,c] Alessandro Silvestri,[d,e] Raquel de Melo Barbosa,[f] Rita Sànchez-Espejo,[g] César Viseras-Iborra,[g,h] Monica Notarbartolo[a] and Serena Riela[i,]**

[a] Dipartimento di Scienze e Tecnologie Biologiche, Chimiche e Farmaceutiche (STEBICEF), Università di Palermo, Viale delle Scienze, Parco d'Orleans II, Ed. 16-17, 90128 Palermo, Italy.

[b] Dipartimento di Fisica e Chimica E. Segrè (DiFC), Università di Palermo, Viale delle Scienze, Parco d'Orleans II, Ed. 17, 90128 Palermo, Italy.

[c] Consorzio Interuniversitario Nazionale per la Scienza e Tecnologia dei Materiali (INSTM), I-50121 Firenze, Italy.

[d] Center for Cooperative Research in Biomaterials (CIC biomaGUNE), Basque Research and Technology Alliance (BRTA), Paseo de Miramon 194, 20014 Donostia-San Sebastián, Spain.

[e] Dipartimento di Scienze Molecolari e Nanosistemi, Università Ca' Foscari Venezia, Via Torino 155, 30170 Venezia Mestre, Italy (current address).

[f] Department of Pharmacy and Pharmaceutical Technology, School of Pharmacy, University of Seville, C/Professor García González 2, 41012, Sevilla, Spain.

[g] Department of Pharmacy and Pharmaceutical Technology, Faculty of Pharmacy, University of Granada, Campus Universitario de Cartuja, 18071 Granada, Spain.

[h] Andalusian Institute of Earth Sciences, CSIC-UGR, 18100 Armilla, Granada, Spain.

[i] Dipartimento di Scienze Chimiche (DSC), Università di Catania, Viale Andrea Doria 6, 95125, Catania, Italy.

Dedicated to Prof. Maurizio Prato in occasion of his retirement.

Total number of pages: 22

Total number of Figures: 16

Total number of Tables: 2

Content

1. Thermogravimetric curves of HNTs-NCD$_s$ nanomaterials……………………………….…...…..…S3

2. FT-IR spectra of HNTs-NCDs$_{a-c}$ nanomaterials synthetized…………………………..….…………....S4

3. XPS survey and deconvoluted spectrum of HNTs-NCDs$_{b-c}$……………………………...…..…....S5

4. Percentage of O, C, and N atoms, as determined by XPS measurements………………..………...S7

5. FT-IR spectra of HNTs-NH_2, HNTs-COOH and HNTs-NCDs**1-2** ……………………...…….S8

6. Thermogravimetric curves of HNTs-NH_2, HNTs-COOH and HNTs-NCDs**1-2** ……..….............S9

7. XPS survey and deconvoluted spectrum of HNTs-NCDs**1-2** and N-doped CDs………………..…S10

8. Percentage of O, C, N and S atoms, as determined by XPS measurements……………..…...…..S12

9. TEM image of N-doped CDs and statistical distribution…………….………………...……..S13

10. HAADF/STEM image and EDX elemental mapping images of HNTs-NCDs**2**….……….…..….S14

11. Absorption spectra of HNTs-NCDs$_{a-c}$; PL emission spectra and Tauc Plot of HNTs-NCDs**2**.…S15

12. PL spectra in solution of HNTs-NCDs$_{b-c}$………………………………………….……..……..S16

13. PL spectra solid state of HNTs-NCDs$_{b-c}$…………………………………………….….…...S17

14. PL spectra in solution of HNTs-NCDs**1** and HNTs-NCDs**2**……………………..….……..…...S19

15. PL spectra solid state of HNTs-NCDs**1** and HNTs-NCDs**2**……………………………..…...S20

16. References…………………………………………………………...…………………S21

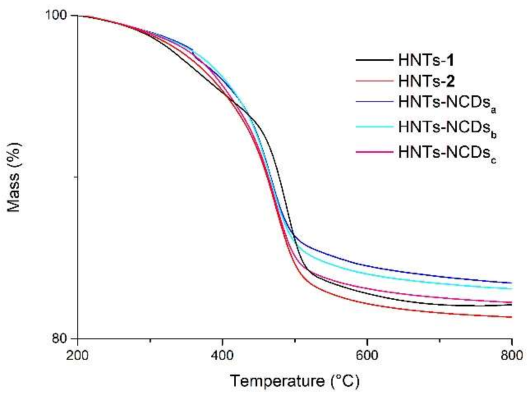

Figure S1. Thermogravimetric curves of HNTs-**1**, HNTs-**2** and HNTs-NCDs$_{a-c}$ based nanomaterials.

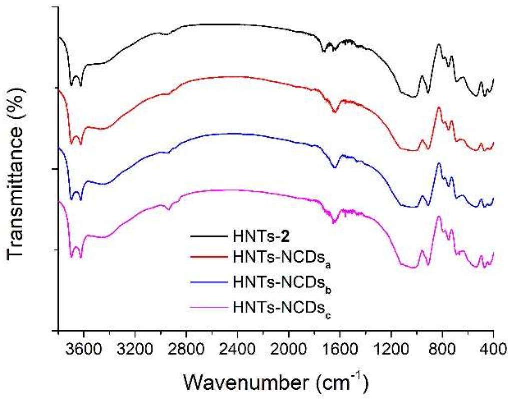

Figure S2. FT-IR spectra of HNTs-**2** and HNTs-$NCDs_{a-c}$

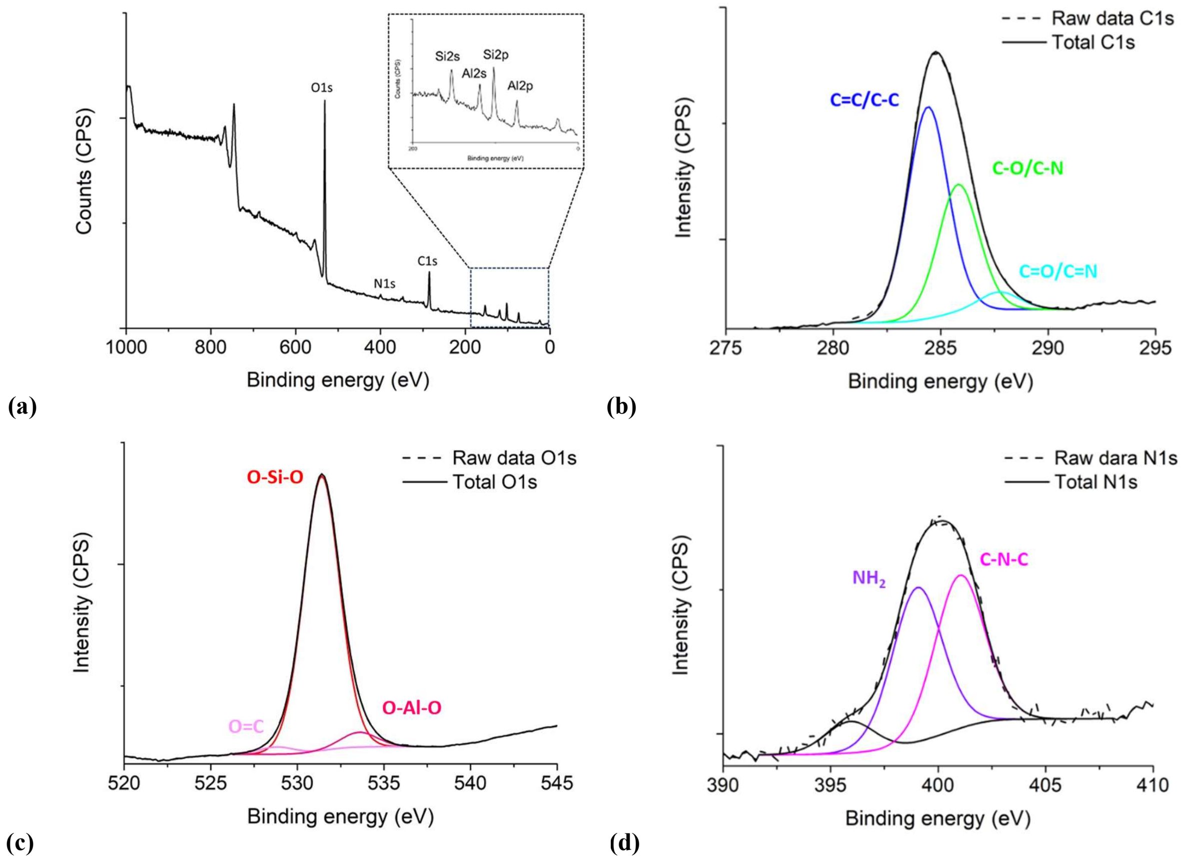

Figure S3. (a) XPS survey of HNTs-$NCDs_b$; (b) deconvoluted C 1s spectrum; (c) deconvoluted O 1s spectrum, (d) deconvoluted N 1s spectrum.

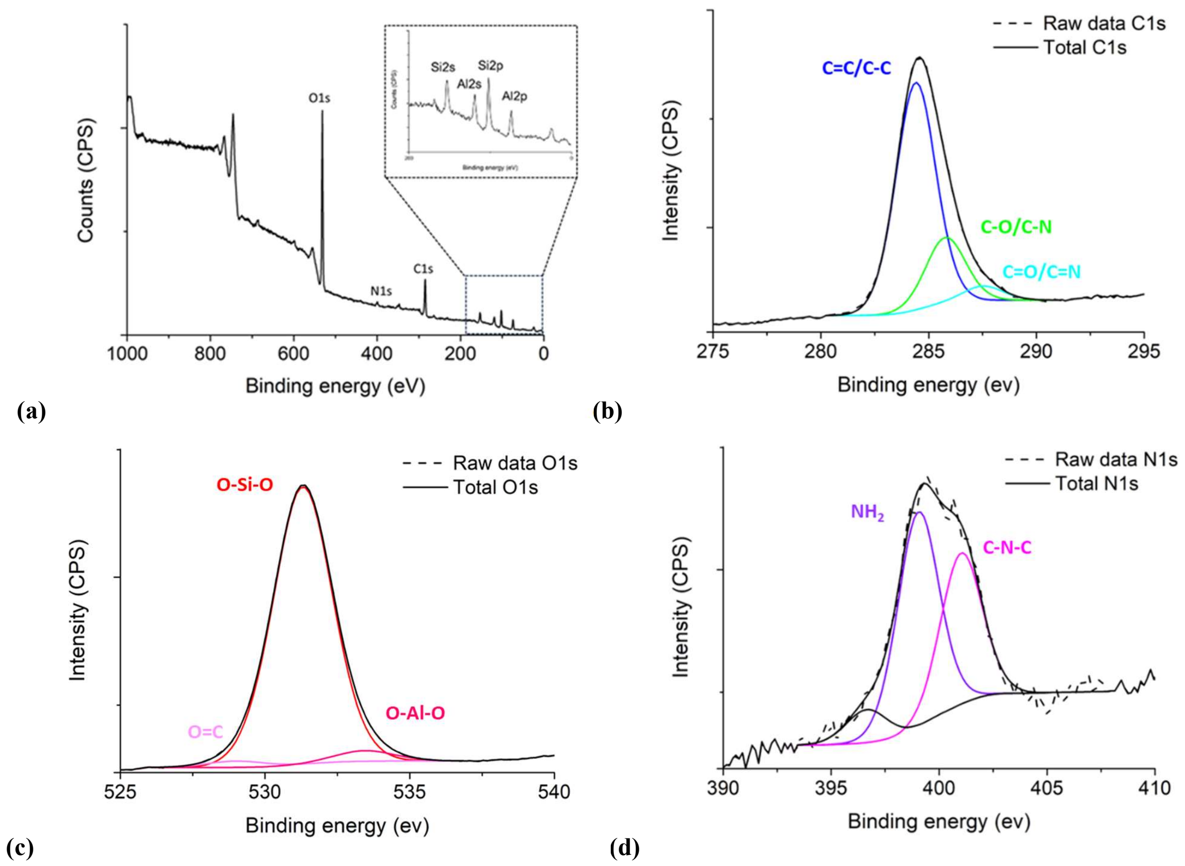

Figure S4. (a) XPS survey of HNTs-NCDs$_c$; (b) deconvoluted C 1s spectrum; (c) deconvoluted O 1s spectrum, (d) deconvoluted N 1s spectrum.

Table S1. Percentage of O, C, N and S atoms, as determined by XPS measurements.

	HNTs-NCDs$_a$	HNTs-NCDs$_b$	HNTs-NCDs$_c$
**Si%**	9.8	12.2	10.3
**Al%**	12.6	12.9	9.5
**C1s %**	17.4	17.8	18.9
**C=C/C-C**	63.6	59.1	73.8
**C-O/C-N**	30.6	35.9	21.4
**C=O/C=N**	5.7	4.9	4.8
**N1s %**	2.3	2.9	2.3
**NH_2**	67.7	50.3	58.9
**C-N-C**	32.3	49.7	41.1
**O1s %**	80.3	79.3	78.8
**O-Si-O**	93.0	92.6	94.3
**O-Al-O**	5.1	5.0	3.6
**O=C**	1.9	2.4	2.1

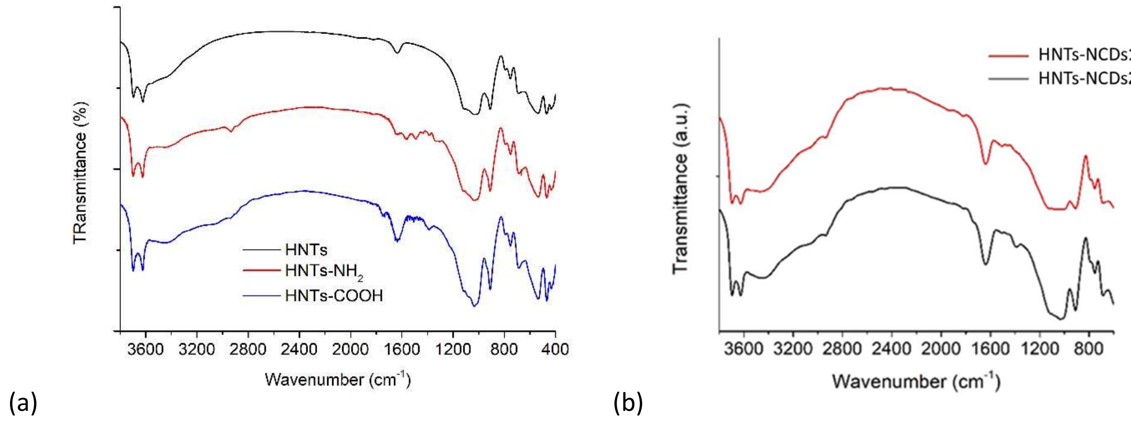

Figure S5 (a) FT-IR spectra of HNTs, HNTs-NH_2 and HNTs-COOH; (b) FT-IR spectra of HNTs-NCDs**1** and HNTs-NCDs**2**

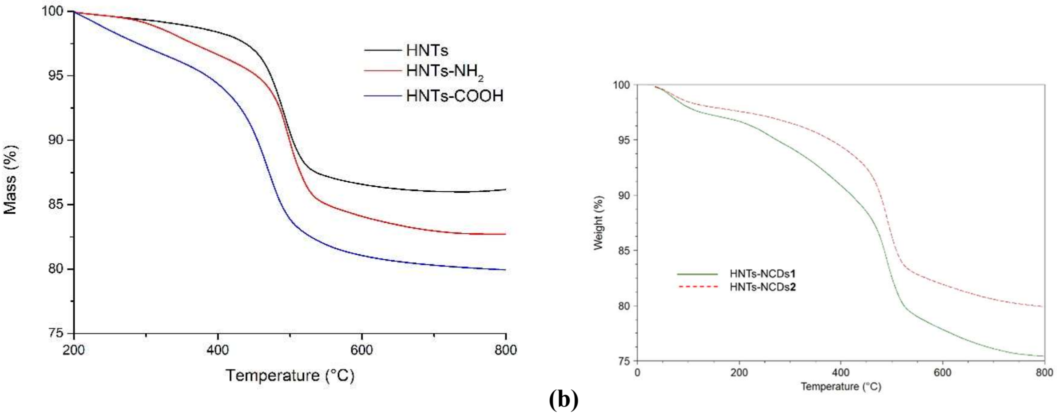

(a) (b)

Figure S6 (a) Thermogravimetric curves of HNTs, HNTs-NH_2 and HNTs-COOH and (b) HNTs-NCDs**1** and HNTs-NCDs**2** based nanomaterials.

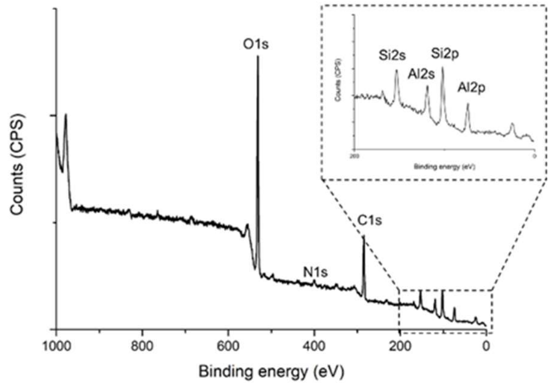
O1s
Si2s
Al2s
Si2p
Al2p
C1s
N1s
Counts (CPS)
Binding energy (eV)
1000
800
600
400
200
0

(a)

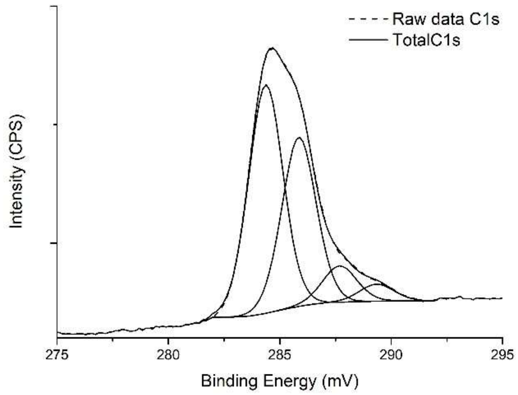
Raw data C1s
TotalC1s
Intensity (CPS)
Binding Energy (mV)
275
280
285
290
295

(b)

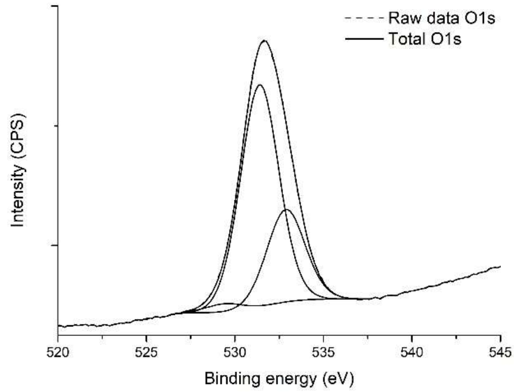
Raw data O1s
Total O1s
Intensity (CPS)
Binding energy (eV)
520
525
530
535
540
545

(c)

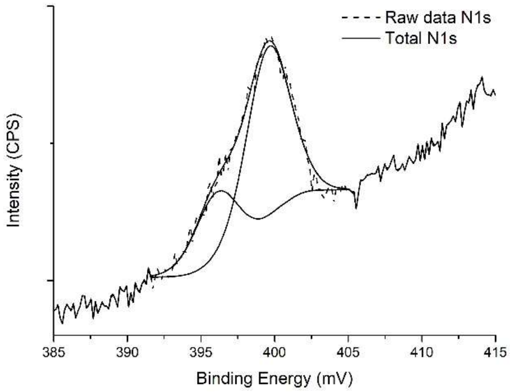
Raw data N1s
Total N1s
Intensity (CPS)
Binding Energy (mV)
385
390
395
400
405
410
415

(d)

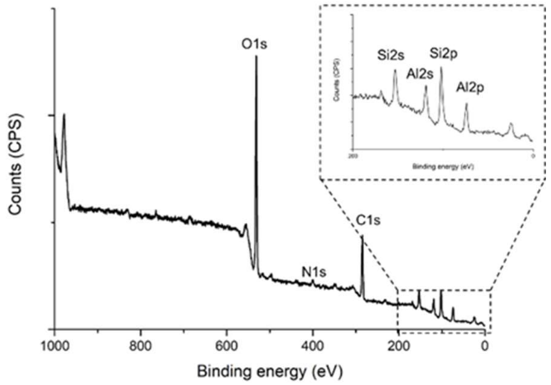
O1s
Si2s
Al2s
Si2p
Al2p
C1s
N1s
Counts (CPS)
Binding energy (eV)
1000
800
600
400
200
0

(e)

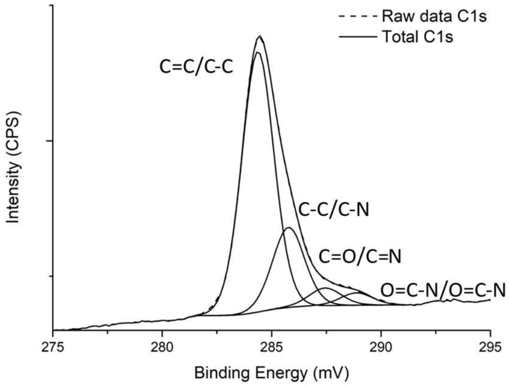
Raw data C1s
Total C1s
C=C/C-C
C-C/C-N
C=O/C=N
O=C-N/O=C-N
Intensity (CPS)
Binding Energy (mV)
275
280
285
290
295

(f)

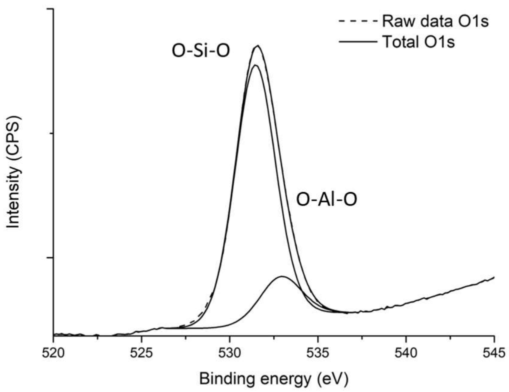
Raw data O1s
Total O1s
O-Si-O
O-Al-O
Intensity (CPS)
Binding energy (eV)
520
525
530
535
540
545

(g)

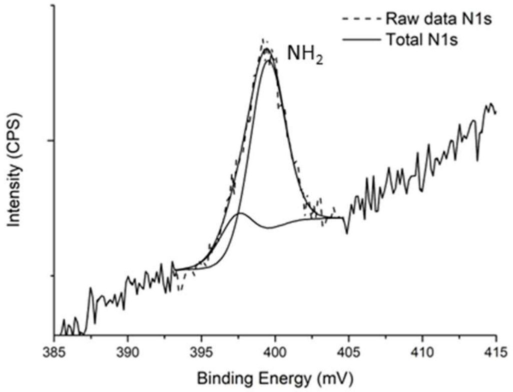
Raw data N1s
Total N1s
NH2
Intensity (CPS)
Binding Energy (mV)
385
390
395
400
405
410
415

(h)

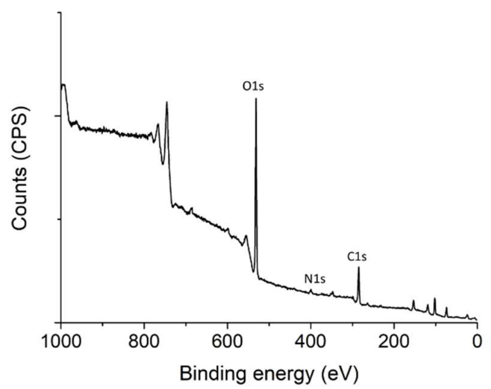

(i)

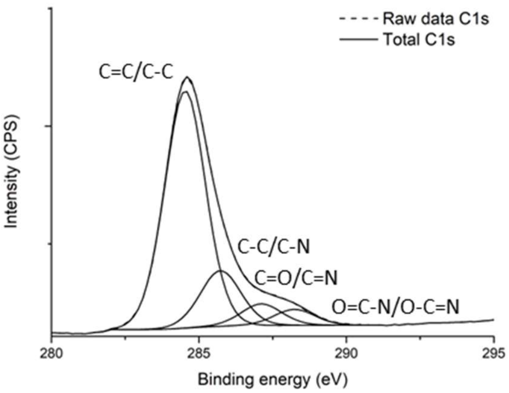

(j)

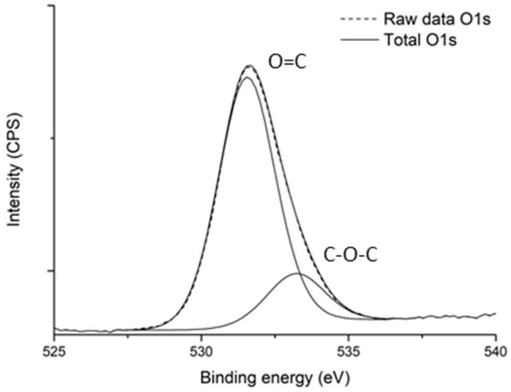

(k)

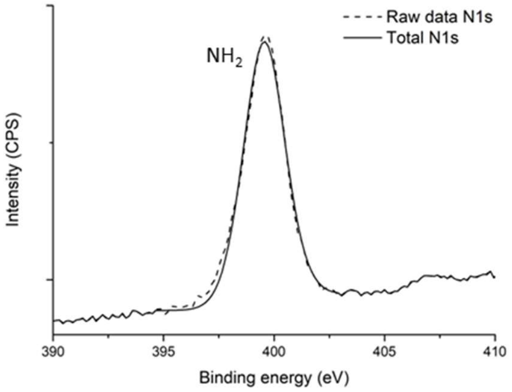

(l)

Figure S7. (a) XPS survey of HNTs-NCDs**1** (b-d) deconvoluted C 1s, O 1s and N1s spectrum of HNTs-NCDs**1** (e) XPS survey of HNTs-NCDs**2** (f-h) deconvoluted C 1s, O1s and N 1s of HNTs-NCDs**2** (i) XPS survey of N-doped CDs (j-l) deconvoluted C 1s, O 1s and N 1s spectrum of N-doped CDs

Table S2. Percentage of O, C, N and S atoms, as determined by XPS measurements

	**N-doped CDs**	**HNTs-NCDs1**	**HNTs-NCDs2**
**Si%**		1.1	1.4
**Al%**		13.3	12.3
**C1s %**	79.0	56.7	65.3
**C=C/C-C**	71.8	50.6	72.3
**C-O/C-N**	16.8	37.5	21.4
**C=O/C=N**	6.5	8.1	3.9
**O=C-N/O-C=N**	4.9	3.8	2.4
**N1s %**	4.4	3.6	1.8
**NH2**	100	70.7	78.7
**O1s %**	16.6	39.7	32.9
**O-Si-O**	-	68.8	86.8
**O-Al-O**	-	28.6	13.2
**O=C**	84.0	2.6	-
**C-O-C**	16.0	-	-

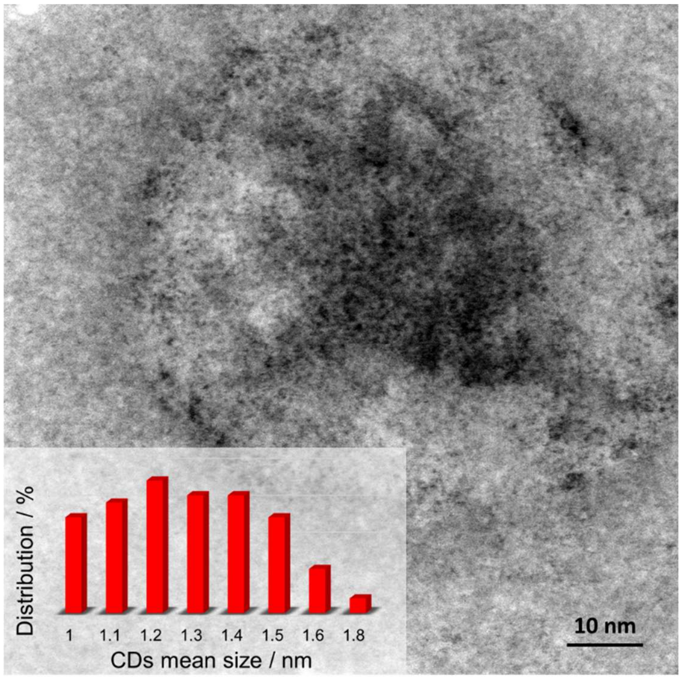

Figure S8 TEM image of pristine N-doped CDs and statistical distribution of the mean size (n = 61).

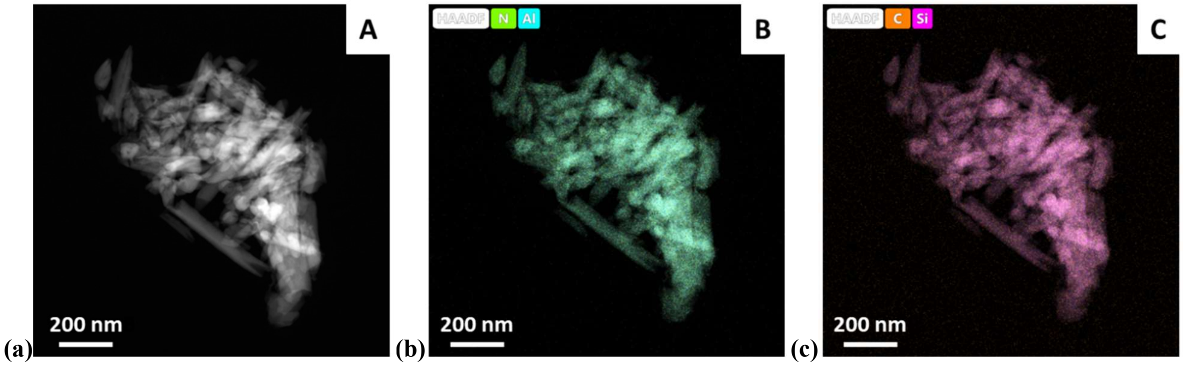

Figure S9 (a) HAADF/STEM image of **HNTs-NCDs2**; (b-c) EDS elemental mapping images.

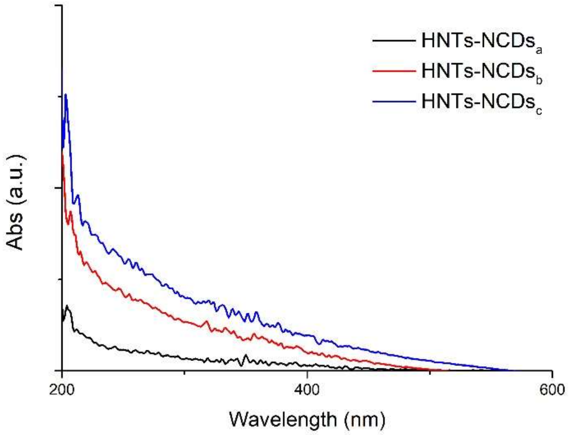

(a)

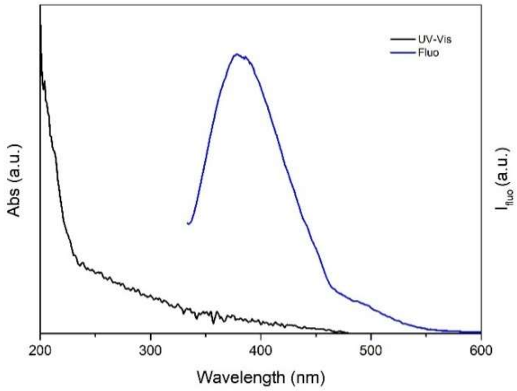

(b)

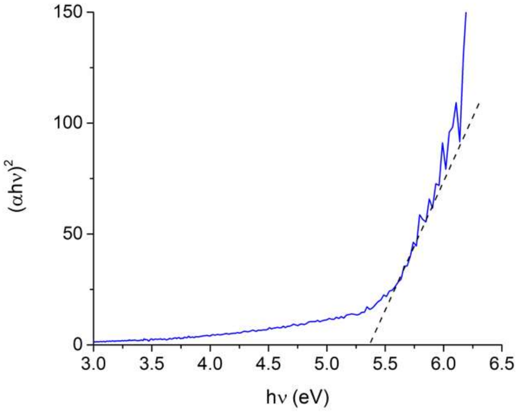

(c)

Figure S10. (a) Absorption spectra of HNTs-NCDs$_{a-c}$; (b) Absorption and PL emission spectra HNTs-NCDs**2**, (c) Tauc plot of HNTs-NCDs**2**.

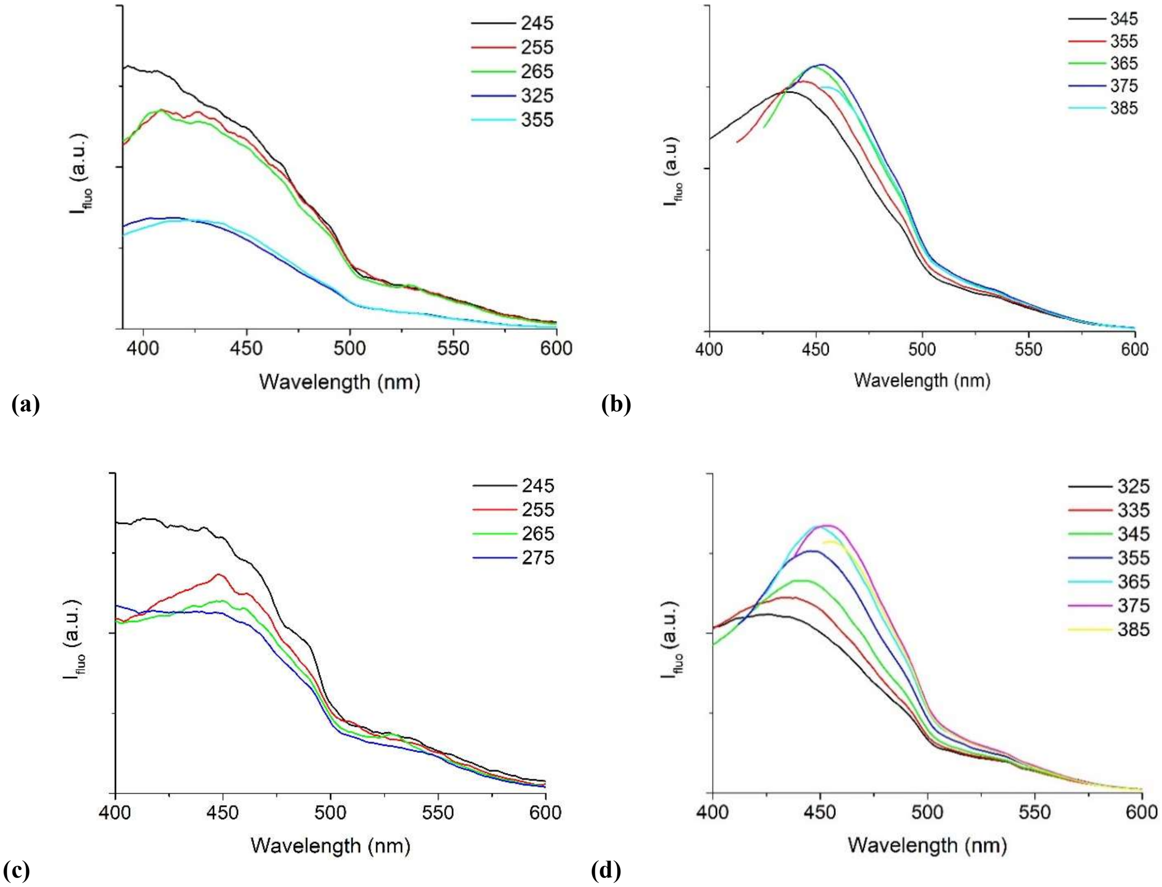

Figure S11. PL spectra in solution of HNTs-NCDs$_{\mathbf{b}}$ (a-b) and HNTs-NCDs$_{\mathbf{c}}$ (c-d) at different excitation wavelength.

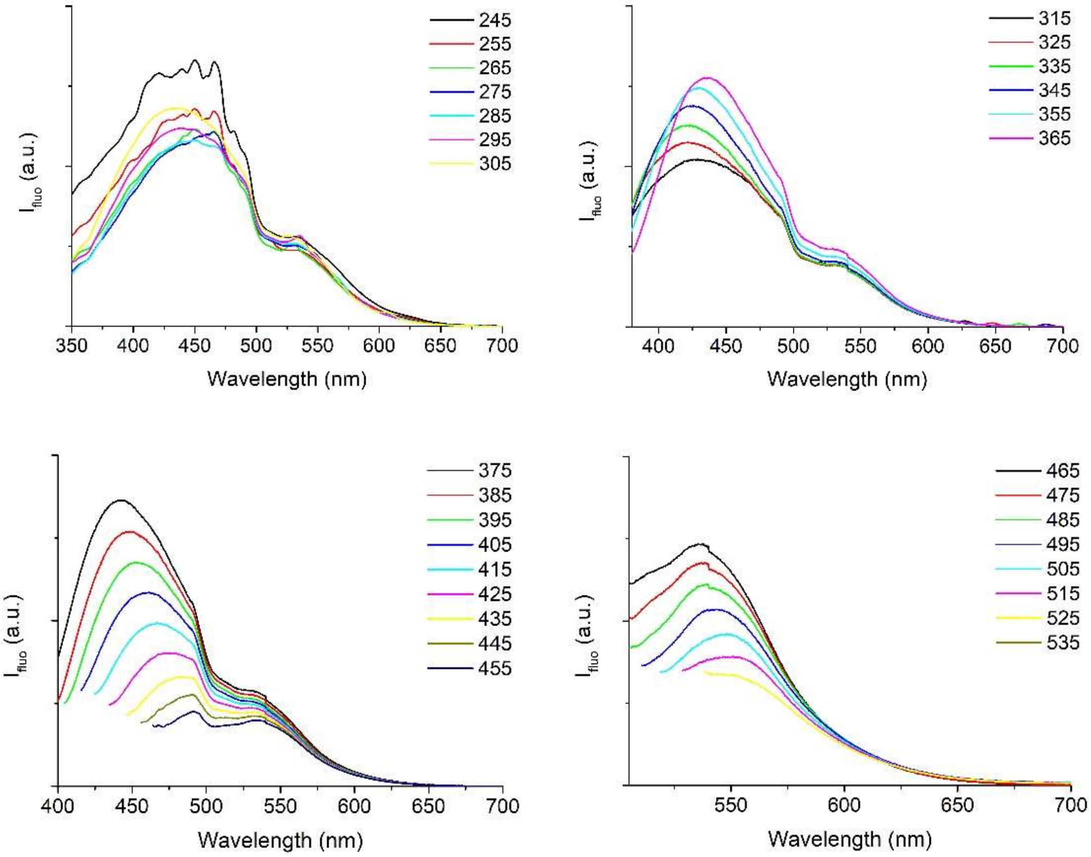

Figure 12. PL spectra solid state of HNTs-NCDs$_b$ at different excitation wavelength.

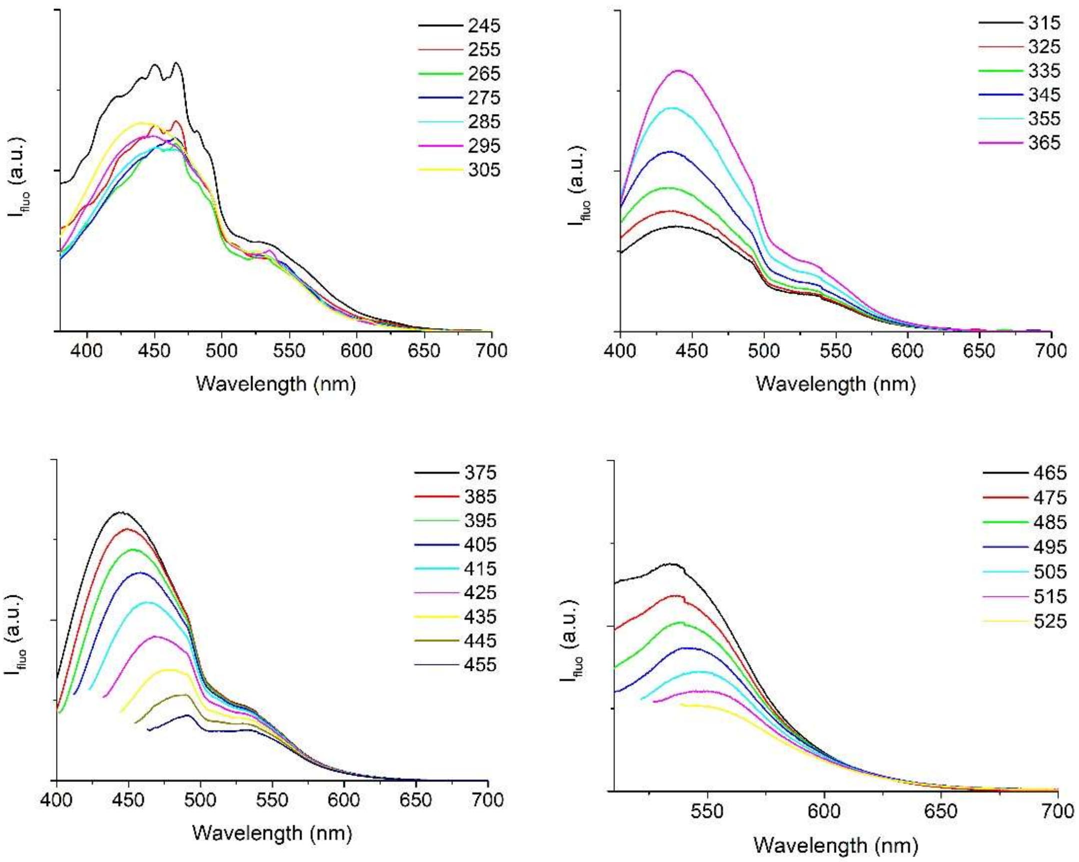

Figure S13. PL spectra solid state of HNTs-$NCDs_c$ at different excitation wavelength.

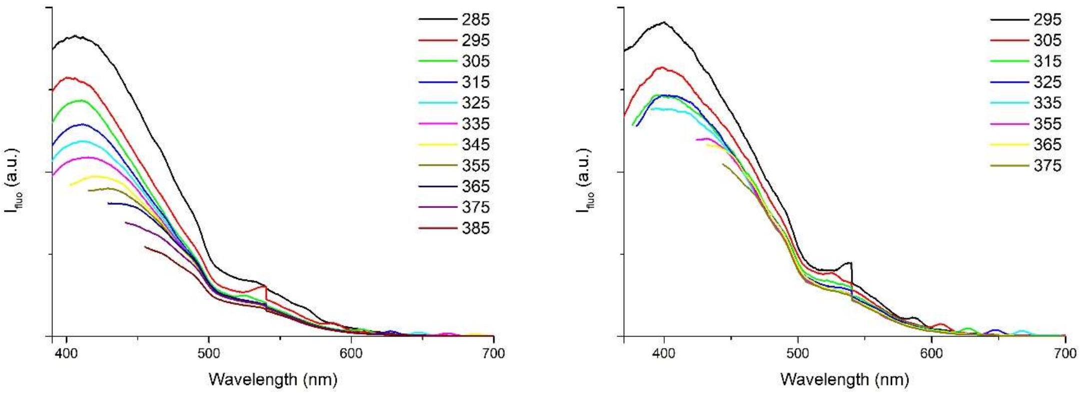

Figure S14. PL spectra in solution of (a) HNTs-NCDs**1** and (b) HNTs-NCDs**2**.

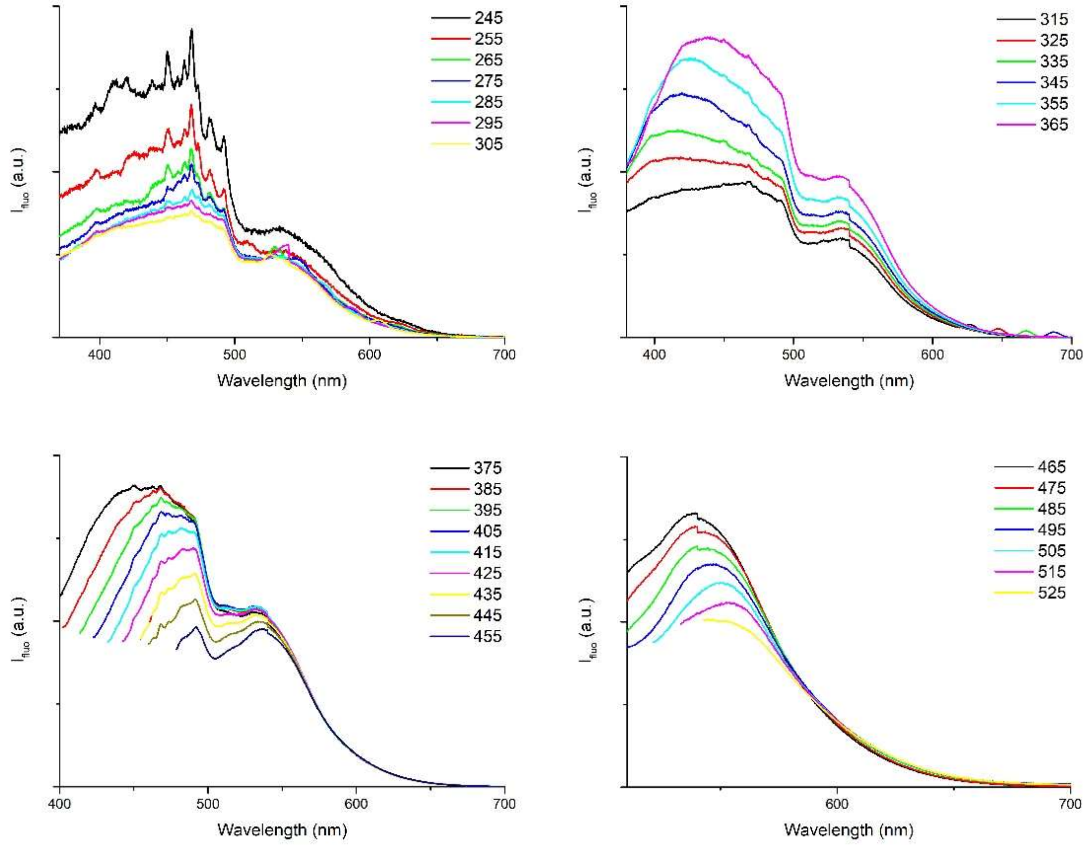

Figure S15. PL spectra solid state of HNTs-NCDs**1** at different excitation wavelength.

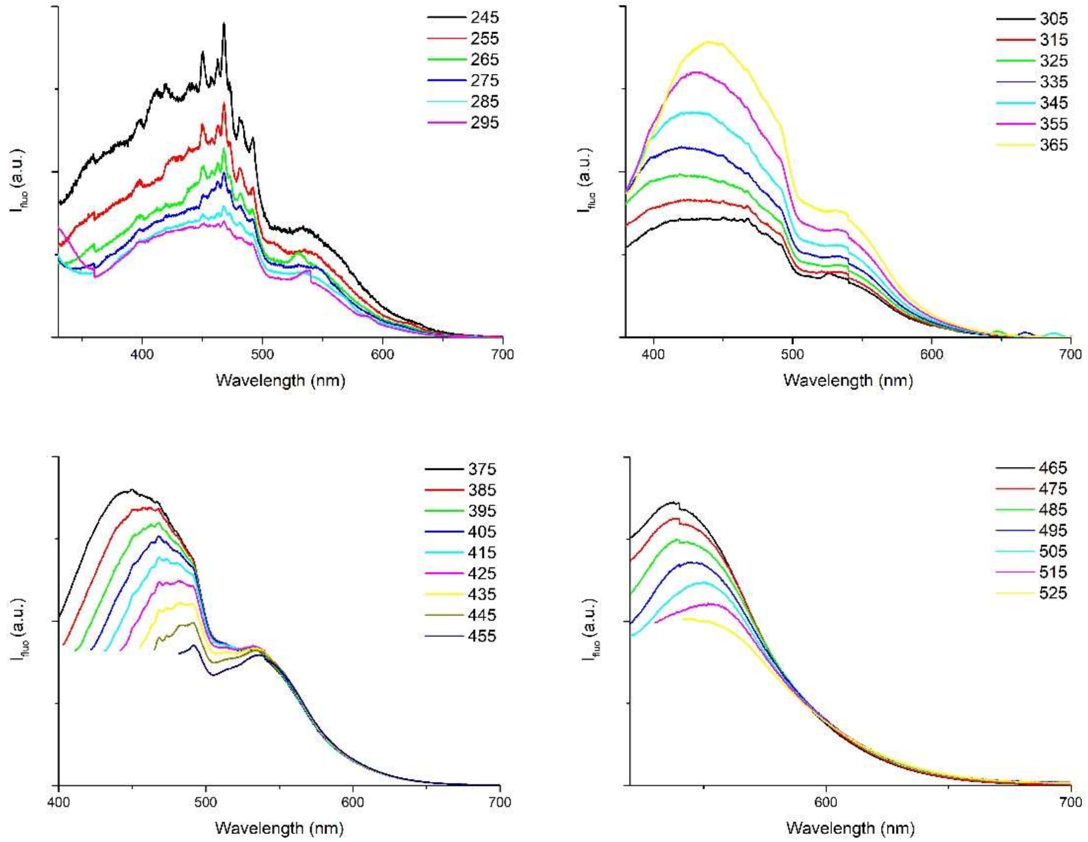

Figure S16. PL spectra solid state of HNTs-NCDs**2** at different excitation wavelength.

References

1. M. Massaro, P. Poma, G. Cavallaro, F. García-Villén, G. Lazzara, M. Notarbartolo, N. Muratore, R. Sánchez-Espejo, C. Viseras Iborra and S. Riela, *Colloids and Surfaces B: Biointerfaces*, 2022, **213**.
2. S. Zhong, C. Zhou, X. Zhang, H. Zhou, H. Li, X. Zhu and Y. Wang, *Journal of Hazardous Materials*, 2014, **276**, 58-65.
3. V. Ramanan, S. H. Subray and P. Ramamurthy, *New Journal of Chemistry*, 2018, **42**, 8933-8942.
4. F. Arcudi, L. Dordevic and M. Prato, *Angewandte Chemie - International Edition*, 2016, **55**, 2107-2112.
